# Deciphering the effects of radiopharmaceutical therapy in the tumor microenvironment of prostate cancer: an in-silico exploration with spatial transcriptomics

**DOI:** 10.7150/thno.99516

**Published:** 2024-10-28

**Authors:** Jimin Hong, Sungwoo Bae, Lara Cavinato, Robert Seifert, Marc Ryhiner, Axel Rominger, Kjell Erlandsson, Moses Wilks, Marc Normandin, Georges El-Fakhri, Hongyoon Choi, Kuangyu Shi

**Affiliations:** 1 Department of Nuclear Medicine, Inselspital, University of Bern, Bern, Switzerland.; 2 Institute of Radiation Medicine, Medical Research Center, Seoul National University College of Medicine, Seoul, South Korea.; 3 Portrai, Inc., 78-18, Dongsulla-gil, Jongno-gu, Seoul, South Korea.; 4 Department of Mathematics, Politecnico de Milano, Milan, Italy.; 5 Institute of Nuclear Medicine, University College London, London, United Kingdom.; 6 Gordon Center of Medical Imaging, Massachusetts General Hospital, Massachusetts, United States of America.; 7 PET Center at Yale School of Medicine, Yale University, New Havens, United States of America.; 8 Department of Nuclear Medicine, Seoul National University Hospital, Seoul, South Korea.

**Keywords:** Radiopharmaceutical/Radioligand Therapy (RPT/RLT), Prostate Cancer, Spatial Transcriptomics, Pharmacokinetic modeling, Dosimetry

## Abstract

Radiopharmaceutical therapy (RPT) is an emerging prostate cancer treatment that delivers radiation to specific molecules within the tumor microenvironment (TME), causing DNA damage and cell death. Given TME heterogeneity, it's crucial to explore RPT dosimetry and biological impacts at the cellular level. We integrated spatial transcriptomics (ST) with computational modeling to investigate the effects of RPT targeting prostate-specific membrane antigen (PSMA), fibroblast activation protein (FAP), and gastrin-releasing peptide receptor (GRPR) each labelled with beta-emitting lutetium-177 (^177^Lu) and alpha-emitting actinium-225 (^225^Ac).

**Methods:** Three ST datasets from primary tissue samples of two prostate cancer patients were obtained. From these datasets, we extracted gene expressions, including FOLH1, GRPR, FAP, and Harris Hypoxia, and estimated the proportions of different cell types—epithelial, endothelial, and prostate cancer (PC) cells—in the corresponding ST spots. We computed the spatiotemporal distribution of each RPT targeting PSMA, FAP, and GRPR at each ST spot by solving the partial differential equation (PDE) using a convection-reaction-diffusion (CRD) model, assuming similar pharmacokinetic parameters across all ligands. A well-established physiologically based pharmacokinetic (PBPK) model was used to simulate the input function in the prostate, carefully calibrated to deliver 10 Gy to the prostate tumor over 20 days. Dosimetry was estimated using the Medical Internal Radiation Dose (MIRD) formalism, applying the dose point kernels (DVK) method. The survival probability was estimated using the linear quadratic model, applied to both beta-emitting RPT labeled with ^177^Lu and ^225^Ac. A modified linear quadratic model was used to estimate the bioeffect of the alpha-emitting RPT.

**Results:** The results demonstrate distinct dose-response and efficacy patterns across ST samples, with FAP-targeted RPT exhibiting limited effectiveness in tumor cell-rich areas compared to PSMA- and GRPR-targeted therapies. GRPR-targeted RPT showed higher resistance in hypoxic regions relative to the other therapies. Additionally, ^225^Ac-labeled RPT was more effective overall than ^177^Lu-labeled RPT, especially in areas with low cancer-cell fraction or high hypoxia. The findings suggest that a combination of ^225^Ac-labeled FAP- and PSMA-targeted RPT offers the best therapeutic strategy.

**Conclusion:** The proposed method, which combines ST and computational modeling to determine the dosimetry and cell survival probability of RPT in the TME, holds promise for identifying optimal personalized RPT strategies.

## Introduction

Radiopharmaceutical therapy (RPT) has recently gained increasing attention in advancing cancer treatment strategies. The primary objective of molecular target-specific RPT is to deliver radiation specifically to target molecule that is expressed on the surface of cancer cells or to the tumor microenvironment (TME), inducing DNA damage and ultimately leading to cell death^1^-^8^. The evolving landscape of RPT prominently centers on two clinically approved cancer targets: prostate-specific membrane antigen (PSMA) and somatostatin receptor 2 (SSTR2). It also encompasses an expanding list of candidates, including fibroblast activation protein (FAP), gastrin-releasing peptide receptor (GRPR), and Integrin αvβ6, with ongoing clinical studies demonstrating their potential in treating various metastatic cancers^8^,^9^. Amongst these, PSMA-targeted RPT has emerged as a crucial treatment option for metastatic castration-resistant prostate cancer (mCRPC)^1^-^3^,^10^,^11^. Notable radioligands employed in PSMA-targeted RPT are labeled with lutetium-177 (^177^Lu) and actinium-225 (^225^Ac)^12^-^15^. While ^177^Lu emits beta particles with an maximum energy of 498 KeV and a tissue range of 0.7 to 2.1 mm, ^225^Ac emits alpha particles with energies ranging from 5.8 to 8.4 MeV and a tissue penetration of 47 to 85 µm^2^. Alpha particles, characterized by high linear energy transfer (LET), can cause severe DNA double-strand breaks (DSBs) regardless of dose rate, cell cycle, or oxygenation status 13,16. Consequently, patients who do not respond to beta-emitting RPT may potentially overcome resistance by receiving alpha-emitting RPT as it induces cellular death even with minimal alpha particle traversal through the cell nucleus^13^,^16^,^17^. Clinical trials are currently underway to validate the efficacy of alpha-emitting RPT^4^,^6^,^18^.

In RPT, an absorbed dose serves as a key physical quantity for translating biological effects such as toxicity and tumor reduction^3^,^6^,^19^. Absorbed dose is defined as the energy absorbed per unit mass of tissue by ionizing radiation and relies on the spatiotemporal distribution of radionuclides which can be estimated by pharmacokinetic models and the types of emitted radiation^20^-^23^. However, the distribution of radiopharmaceuticals within tumor tissue may exhibit significant heterogeneity due to variations in local physiology and the biochemical properties of radiopharmaceuticals^24^-^26^. Moreover, the TME inherently contributes to heterogeneity in absorbed dose and, consequently, biological effects^19^,^22^. The TME comprises diverse cellular components, including cancer-associated fibroblasts (CAFs) and endothelial cells of the neo-vasculature, which can influence the processes of pro-/anti-tumorigenic factors characterized by acidity, altered metabolism, and hypoxia^27^,^28^. For instance, hypoxia has been identified as one of the resisting factors in RPT^29^. In addition, the expression levels of target molecules, such as PSMA in prostate cancer cells, varies depending on the specific location of the cancer cells within the TME^30^. Hence, understanding these cellular-level variations is crucial, especially for alpha-emitting RPT, as it requires precise dose assessment at the microscopic scale due to its limited radiation penetration depth^25^,^26^.

Spatial transcriptomics (ST) is a cutting-edge technique that revolutionizes our understanding of tumor heterogeneity, providing genome-wide expression data with spatial information at the resolution of dozens of cells^31^-^34^. It combines a barcode-based RNA capture method with high-throughput RNA sequencing to reconstruct spatially resolved transcriptomic information^31^. Hence, ST offers a unique opportunity to study the heterogeneity of gene expression patterns within tumors and the complex interactions between various cell types within the TME^35^-^37^. In this regard, incorporating high resolution molecular information derived from ST, such as PSMA expression and hypoxia gene signatures, and the distributions of various cell types into RPT dosimetry simulation enables a more precise assessment of therapeutic response and facilitates the optimization of treatment strategies^29^,^38^-^40^.

In this study, our objective was to develop a comprehensive method for modeling the dosimetry and biological effects of RPT at the cellular level, with a focus on addressing microscopic heterogeneities. To achieve this, we employed ST and pharmacokinetic models, focusing on RPT with ^177^Lu and ^225^Ac-labelled ligands. We leveraged ST datasets of prostate cancer (PC) tissue and extracted the spatial distribution of various gene expressions associated with TME such as a gene coding the target for RPT and hypoxia-related gene signature as well as cell types (epithelial, endothelial, and malignant cells) within the microscopic tumor tissue^39^. Subsequently, we computed the spatiotemporal biodistribution of each radiopharmaceutical using a three-compartment (interstitial, bounded, internalized) convection-reaction-diffusion (CRD) model, incorporating various ST-based feature maps. The absorbed dose map was then calculated using dose-point kernel (DPK) method, following the Medical Internal Radiation Dose (MIRD) formalism. The resulting dose distribution was analyzed using the dose-volume histogram (DVH). Finally, we assessed the biological effect by calculating the cell survival probability using a linear quadratic (LQ) model and modified LQ model, which accounts for the dose and hypoxia map derived from ST datasets^40^-^43^.

## Results

### *In silico* modeling with spatial transcriptomics

**Figure 1:**
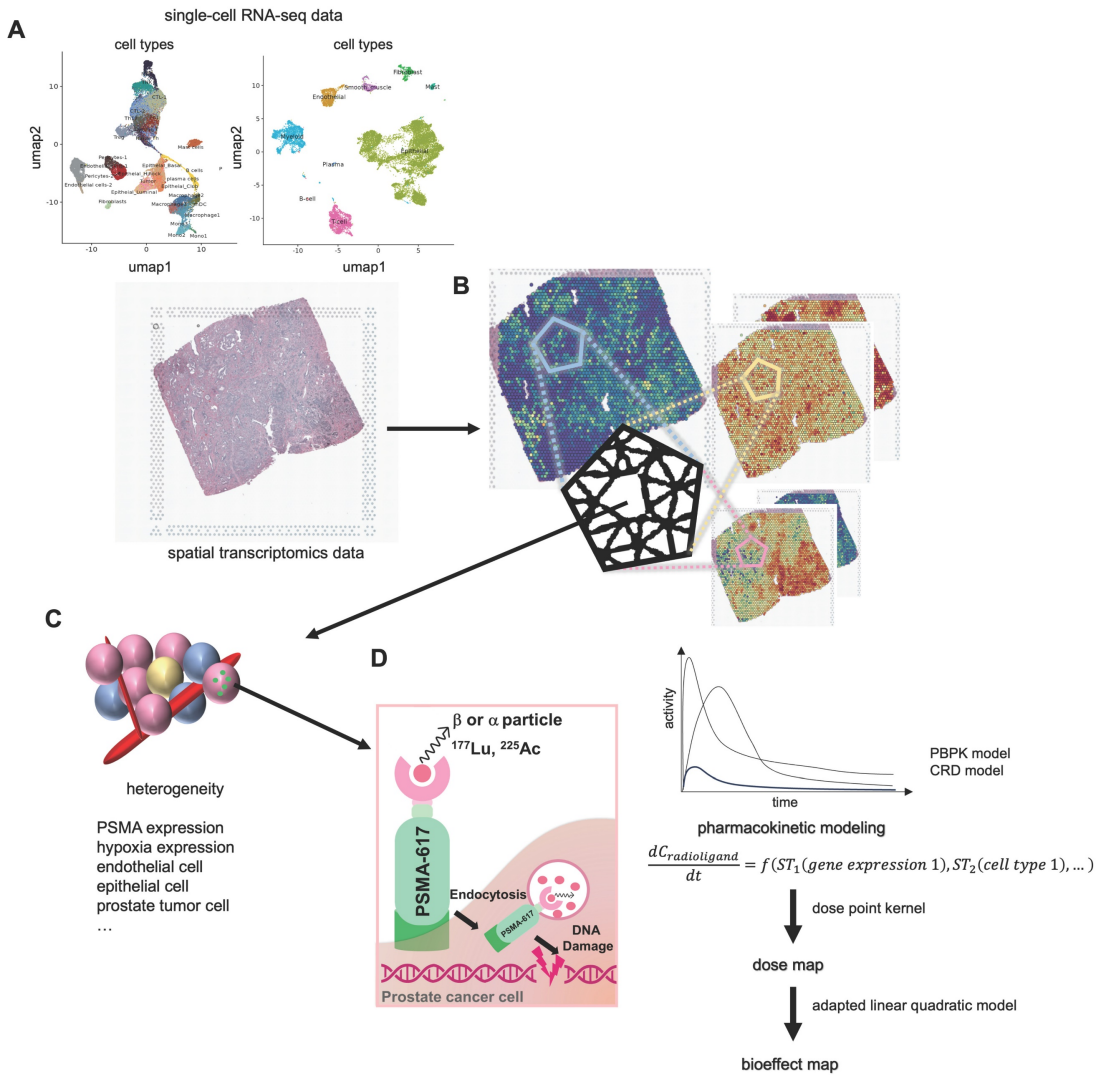
** Schematics of dose and bioeffect modeling on based spatial transcriptomics (ST). (A)** The distributions of various cell types (endothelial, epithelial, tumor, …) are determined using CellDART utilizing single-cell RNA-seq data (scRNA-seq) and ST. **(B)** The tissue composition is subsequently clarified by delineating the spatial distribution of distinct cell types and gene expressions. **(C)** Each ST spot comprises a small number of cells, each contributing to the heterogeneity of the tumor microenvironment (TME). **(D)** The prostate-membrane specific antigen (PSMA, green) is expressed on the surface of the prostate cancer cell (magenta). Radioligands labeled with ^177^Lu or ^225^Ac are primarily utilized for PSMA-targeted radiopharmaceutical therapy (RPT), emitting beta and alpha particles, respectively. Simulating the time-activity curve (TAC) of the radioligand in each spatial transcriptomics (ST) spot involves solving a partial differential equation (PDE) representing a pharmacokinetic model. Dose and its bioeffect are modeled using a dose point kernel (DPK) and a modified linear quadratic (LQ) model. This allows for the assessment of RPT efficacy in each ST spot.

Our methodology for determining the dose and cell survival probability of RPT with the ST is outlined in the Methods section, accompanied by a schematic summary of study design** (Figure 1)**. Initially, we utilized the CellDART^44^ algorithm to estimate the spatial distribution of cell types in TME, integrating ST data with single-cell RNA-seq data (scRNA-seq) obtained from PC tissues. Subsequently, utilizing the spatial distribution maps of various cell types and gene expression within the TME, we simulated the spatial-temporal distribution of [^177^Lu]Lu- and [^225^Ac]Ac-PSMA-ligand over a 14-day period targeting 10 Gy by solving partial differential equations (PDEs) representing the three-compartment CRD-based model. Absorbed dose map was then estimated by integrating activities solved by PDEs and employing the DPK method. Additionally, we quantified the bioeffect of the corresponding dose map by calculating cell survival probability using LQ models. The feasibility of the model was tested in prostate cancer tissues for PSMA-targeted RPTs. It was also tested for FAP and GRPR, which are other emerging targets for RPT in cancer.

### Spatial distribution of endothelial cells, tumor cells, hypoxia, and PSMA binding density

**Figure 2:**
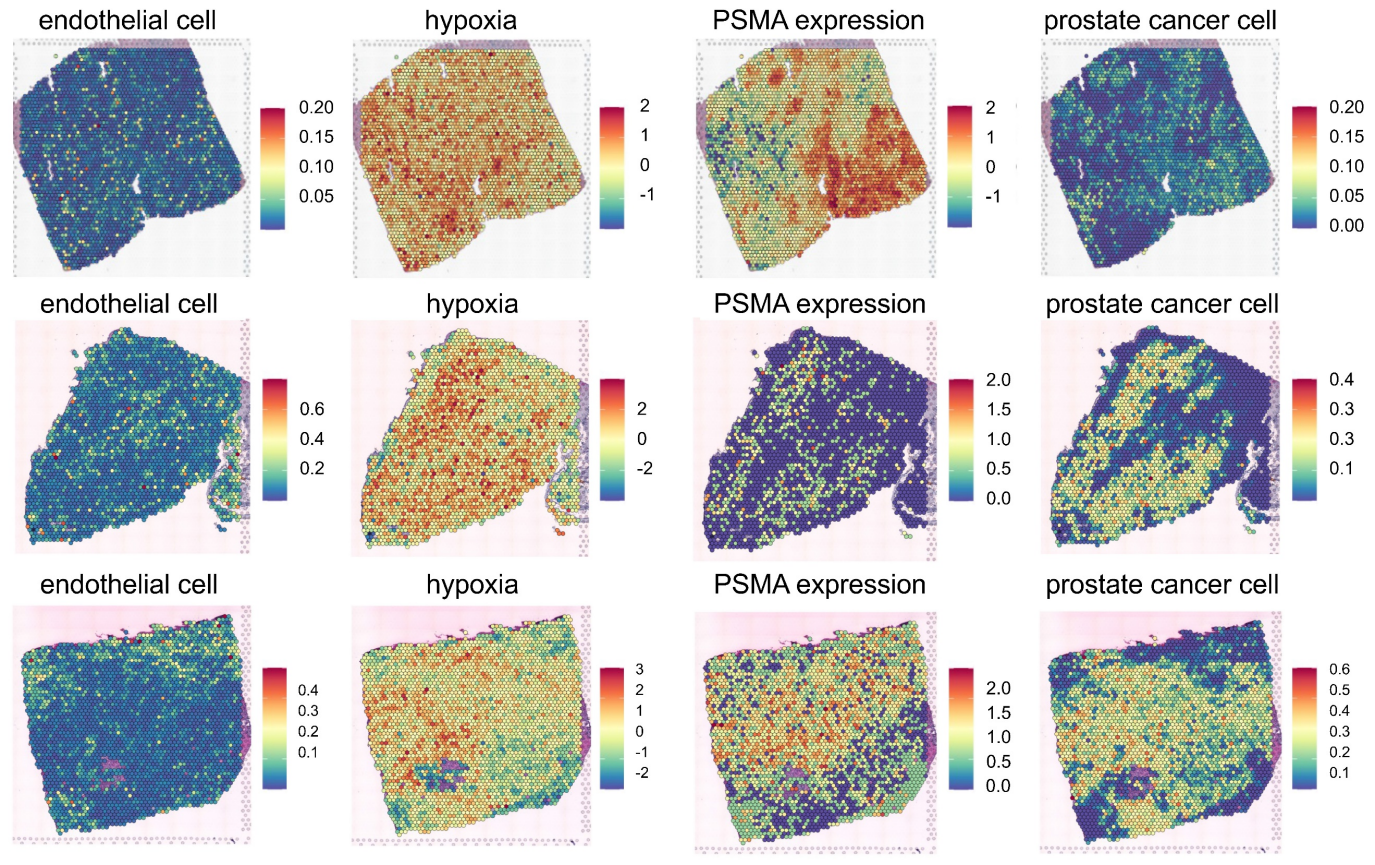
** Spatial Transcriptomics (ST) mapping of prostate cancer (PC) tissue (PC1 - top, PC2-middle, PC3 - bottom).** Each ST spot covers cell types/gene expression within a diameter of 55 micrometers, with a distance of 100 micrometers between the spots. The fractions of different cell types (endothelial and prostate cancer cells) in the corresponding ST locations are mapped. The color scale for each cell type represents the fraction of that specific cell type in each ST spot, with all cell type fractions summing up to 1, reflecting the cellular composition in each ST location. The Hypoxia map shows the distribution of the hypoxia-related gene scores, Harris Hypoxia, while the PSMA density map illustrates the spatial expression of the PSMA encoding gene, FOLH1. The color scale for both Hypoxia and PSMA density map represents the values after normalization and log-transformation of raw counts of each Harris Hypoxia and FOLH1 expressions.

Three PC tissues were utilized for* in silico* modeling of PSMA-targeted RPTs **(Figure 2)**. The first PC tissue (PC1) was extracted from a stage IV prostate cancer patient presenting with acinar cell carcinoma and the international society of urological pathology (ISUP) grade of 2 or 3. The second and third PC tissues (PC2 and PC3) were both obtained from treatment-naïve prostate cancer patients with adenocarcinoma and the ISUP grade of 3. ST data revealed heterogeneous spatial distributions of PSMA expression*,* hypoxia, endothelial cells, and PC cells***,*** while distributions of the other cell types were also identified **(Figure S1A-S1C)**. The capture area for all three ST datasets illustrated **(Figure 2)** is 6.5 x 6.5 mm. There are a total of 4992 total spots per capture area and each spot is 55 µm in diameter with a 100 µm center to center distance between spots. *FOLH1* expression is used as surrogate for PSMA expression, and hypoxia distribution is estimated using gene sets defined by the *Harris hypoxia* signature^39^. Three PC tissues showed distinctive spatial patterns of gene expression and cell types **(Figure S2B-S2D)**. In PC1, distinct proportions of ST spots encapsulated vascular structures (5.35%), hypoxic regions (9.59%), and high PSMA density regions (56.49%). PC2 exhibited different percentages, registering at 25.42%, 15.08%, and 27.14%, respectively. Meanwhile, PC3 reported percentages of 12%, 14.22%, and 41.85%. The dissimilarity between the distributions of vessel and PSMA density maps measured by Kullback-Leibler divergence (KLD) was 0.20, 0.95, and 0.25 for each respective dataset. Similarly, the dissimilarity between the distributions of hypoxia and PSMA density maps exhibited values of 0.33, 1.13, and 0.89 for the three datasets, respectively.

### Dose distribution in PSMA-targeted RPTs

**Figure 3:**
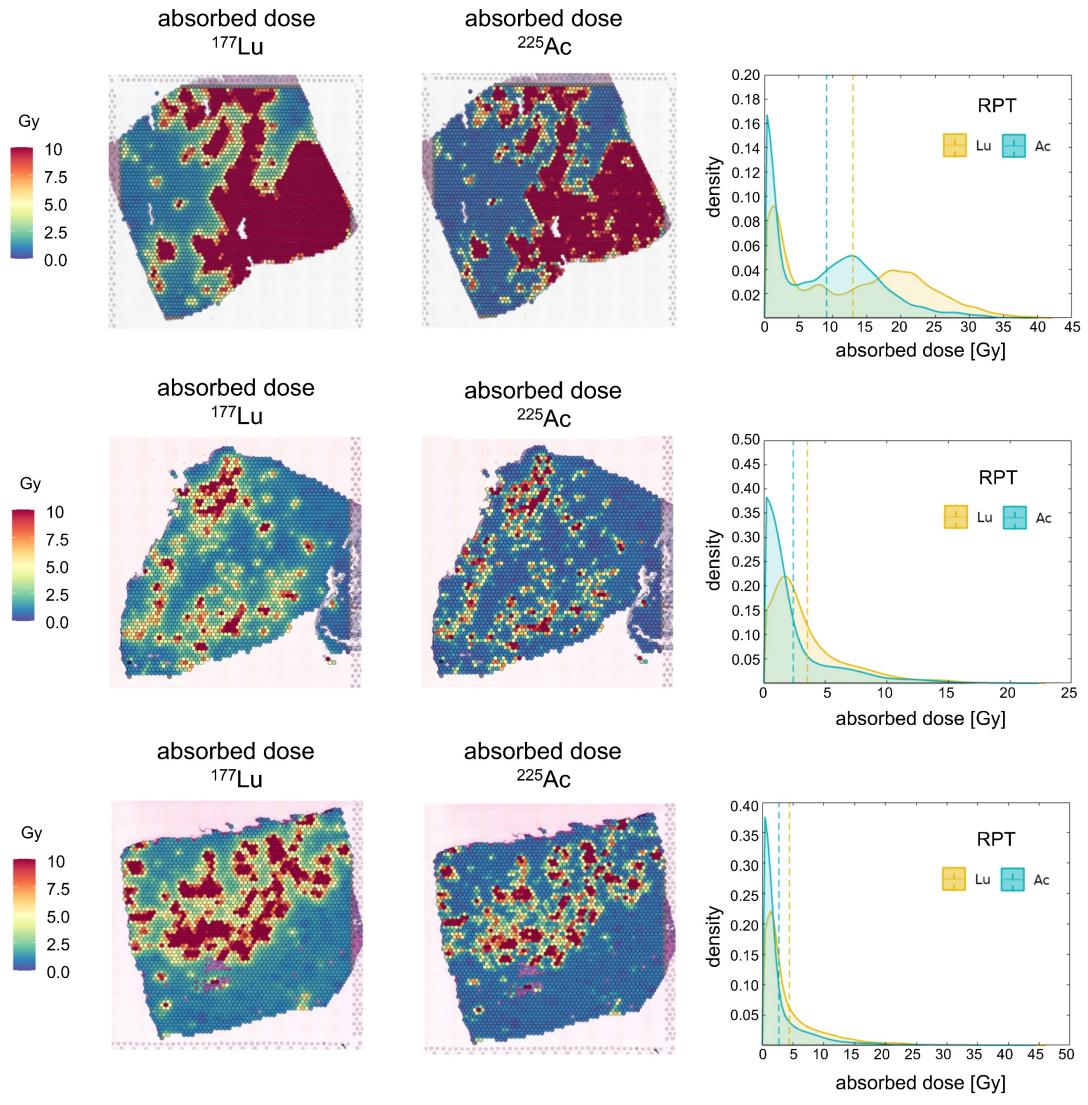
** Absorbed Dose of [^177^Lu]Lu- and [^225^Ac]Ac-PSMA-targeted RPT in three prostate cancer tissues.** The spatial distribution of D is depicted derived from each ^177^Lu- (left) and ^225^Ac-PSMA-targeted RPT simulation (middle). Kernel Density Estimation (KDE) demonstrates the distribution of D (right) in the tumor cell-abundant area for ^177^Lu-PSMA-targeted RPT (yellow) and ^225^Ac-PSMA-targeted RPT (turquoise), defined by a cutoff of 0.01 in the prostate cancer map. The dashed line indicates the average values of D.

The spatiotemporal distributions of radioactivity corresponding to [^177^Lu]Lu- and [^225^Ac]Ac-PSMA-ligand were simulated based on the derived maps of cancer cells and gene expression. The resulting absorbed dose maps were derived, and kernel density estimation (KDE) was applied to visualize the probability density distribution of the dose in the tumor cell-abundant region **(Figure 3)**, while the DVH analysis provided insights into the distribution of absorbed doses in entire region **(Figure S2A)**. Across all three tissue simulations, [^225^Ac]Ac-PSMA-ligand, on average, deposited a lower dose in the target area compared to [^177^Lu]Lu-PSMA-ligand. Specifically, in PC1, the average absorbed doses were 12.97 Gy and 9.08 Gy for [^177^Lu]Lu-PSMA-ligand and [^225^Ac]Ac-PSMA-ligand, respectively. For PC2, these values were 3.54 Gy and 2.39 Gy, and for PC3, 4.60 Gy and 2.83 Gy. Consequently, the KDE exhibited a steeper curve centered around zero for [^225^Ac]Ac-PSMA-ligand compared to [^177^Lu]Lu-PSMA-ligand.

Overall, elevated doses were delivered to ST spots with a higher concentration of PC cells. However, the dose plateaued after reaching a certain threshold in PC1 and PC3 **(Figure S3A)**. For example, in PC1, when examining tumor cell-depleted regions, we found a positive relationship between dose and tumor cell fraction in for both ^177^Lu (Spearman ρ = 0.304, p < 0.0001) and ^225^Ac (Spearman ρ = 0.305, p < 0.0001). However, in tumor cell-abundant regions, we observed no significant relationship between dose and PC cell fraction in neither [^177^Lu]Lu- nor [^225^Ac]Ac-PSMA-ligand. Conversely, in PC2, spots with a higher tumor cell fraction consistently received a higher dose. In both tumor cell-depleted region and tumor cell-abundant region, ^177^Lu and ^225^Ac showed positive relationship between the tumor cell fraction and the absorbed dose. Notably, ^177^Lu showed stronger correlation (tumor cell-depleted: 0.348, p<0.0001, tumor cell-abundant: 0.416, p<0.0001), compared to ^225^Ac (tumor cell-depleted: 0.183, p<0.0001, tumor cell-abundant: 0.364, p<0.0001).

### Cell survival probability analysis in PSMA-targeted RPTs

**Figure 4:**
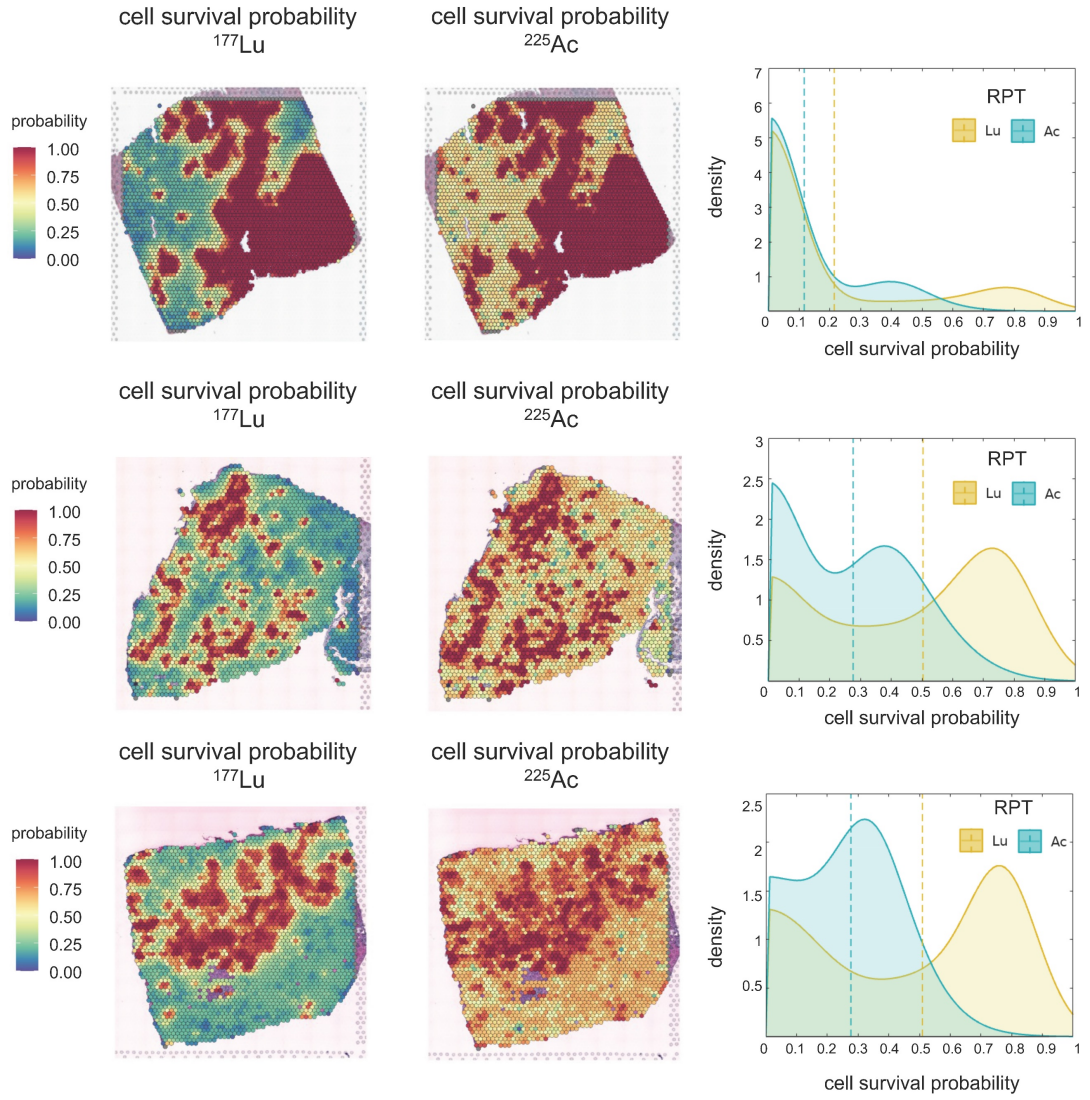
** Cell Survival Probability (Ps) of [^177^Lu]Lu- and [^225^Ac]Ac-PSMA-targeted RPT in three prostate cancer tissues.** The spatial distribution of Ps is depicted derived from each [^177^Lu]Lu- (left) and [^225^Ac]Ac-PSMA-targeted RPT (middle) simulation. Kernel Density Estimation (KDE) demonstrates the distribution of Ps (right) in the tumor cell-abundant area for [^177^Lu]Lu- (yellow) and [^225^Ac]Ac-PSMA-targeted RPT (turquoise), defined by a cutoff of 0.01 in the prostate cancer map. The dashed line indicates the average values of Ps.

Cell survival probability within tumor cell-abundant regions was estimated, and the density distribution was visualized using KDE** (Figure 4)** in simulations of both [^177^Lu]Lu- and [^225^Ac]Ac-PSMA-targeted RPT. Notably, distinct peaks in cell survival probability were observed in both cases, with [^177^Lu]Lu- exhibiting a more pronounced difference between the means of these peaks compared to [^225^Ac]Ac-PSMA-targeted RPT. This observation suggests that [^225^Ac]Ac-PSMA-targeted RPT induces a more selective response in cell survival probability, with cells belonging to either peak more likely to be killed. Conversely, in [^177^Lu]Lu-PSMA targeted RPT, regions corresponding to the second peak exhibit a higher survival probability, indicating greater resistance to RPT.

The simulation result suggests that within the TME, tumor cell-abundant regions are more responsive to RPT, whereas tumor cell-depleted regions tend to exhibit resistance following RPT. Notably, ^225^Ac-labeled RPT demonstrates superior efficacy in reducing tumor cell survival probability especially in tumor cell-depleted areas, compared to ^177^Lu-labeled RPT. The average cell survival probabilities within tumor cell-abundant regions were determined to be 0.21 and 0.11 in PC1, 0.50 and 0.27 in PC2, and 0.48 and 0.27 in PC3 for [^177^Lu]Lu- and [^225^Ac]Ac-PSMA-ligand, respectively. In tumor cell-depleted region, the corresponding averages were 0.51 and 0.31 in PC1, 0.74 and 0.42 in PC2, and 0.60 and 0.31 for [^177^Lu]Lu- and [^225^Ac]Ac-PSMA-ligand, respectively. With an increase in tumor cell fraction in ST spots, the cell survival probability decreased **(Figure S3B).**

### Influence of PSMA receptor density and hypoxia in PSMA-targeted RPTs

**Figure 5:**
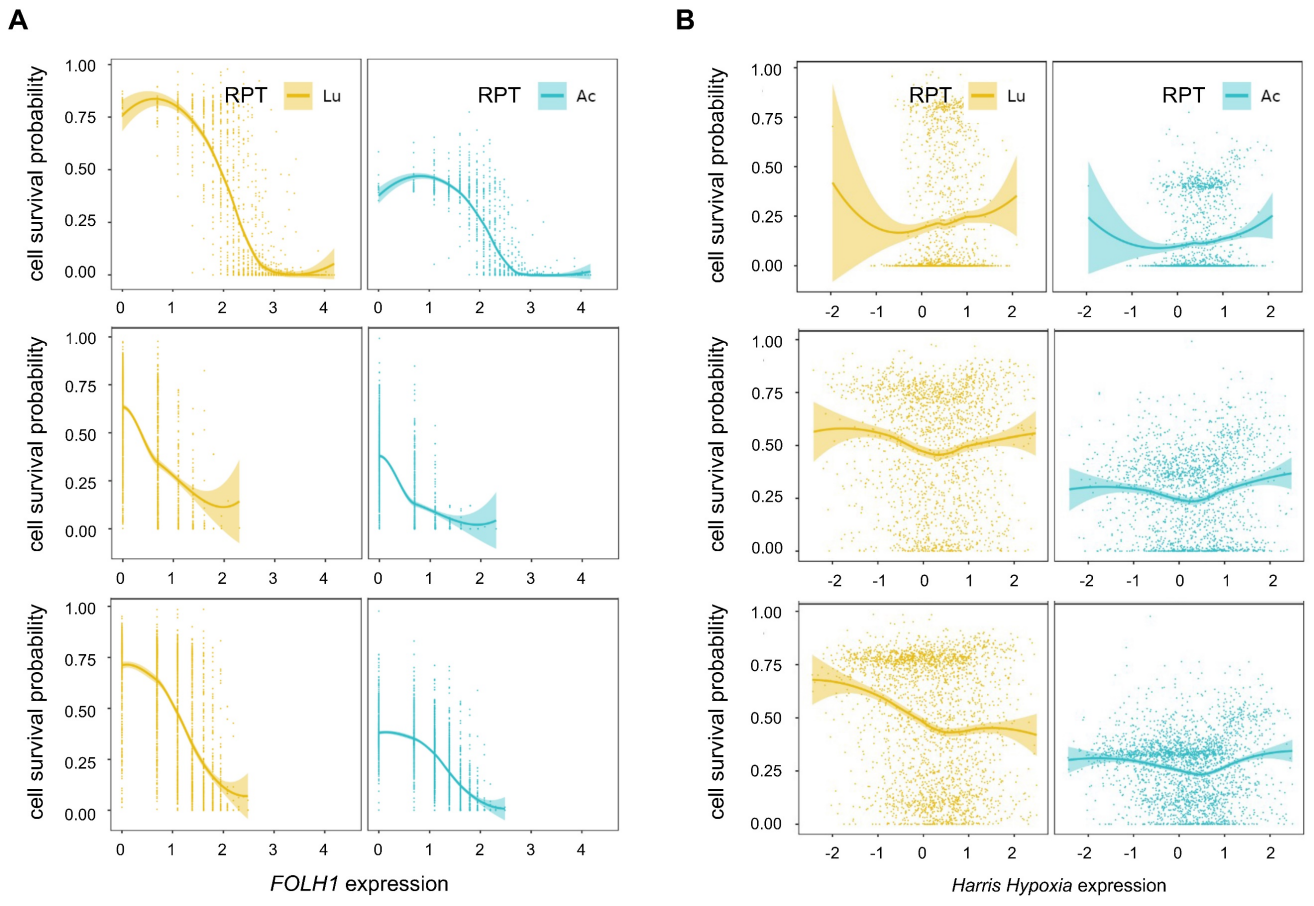
** Correlation between influencing factors of PSMA-RPT and cell survival probability in the tumor cell-abundant regions of three prostate cancer tissues (PC1-top, PC2-middle, PC3-bottom). (A)** Correlation between PSMA density and cell survival probability **(B)** Correlation between hypoxia and cell survival probability. The solid line represents the estimation based on locally estimated scatterplot smoothing (LOESS) local regression fitting, and the area around the solid line indicates the 95% confidence of the fitting lines.

The PSMA density exhibited a strong negative correlation with cell survival probability in all tissue samples, particularly in tumor cell-abundant regions in both [^225^Ac]Ac- (PC1: Spearman ρ = -0.76, p<0.0001, PC2: Spearman ρ = -0.68, p<0.0001, PC3: Spearman ρ = -0.66, p<0.0001) and [^177^Lu]Lu-PSMA-targeted RPT simulation (PC1: Spearman ρ = -0.76, p<0.0001, PC2: Spearman ρ = -0.59, p<0.0001, PC3: Spearman ρ = -0.64, p<0.0001) **(Figure 5A)**. Overall, Compared to [^177^Lu]Lu-, [^225^Ac]Ac-PSMA-targeted RPT demonstrated lower resistance to RPT in areas with low PSMA density, resulting in a decreased cell survival probability. The influence of PSMA density on tumor cell killing effect was slightly more pronounced in [^177^Lu]Lu- compared to [^225^Ac]Ac-PSMA-targeted RPT in regions with low PSMA density. This suggests that the efficacy of RPT was more sensitive to PSMA density in ^177^Lu than in ^225^Ac.

Hypoxia showed a weak positive correlation with cell survival in both [^177^Lu]Lu- and [^225^Ac]Ac-PSMA targeting RPT within the tumor cell-depleted region in general **(Figure 5B)**. However, a weak or no significant correlation was observed in the tumor cell-abundant region. Despite hypoxia being traditionally regarded as a resistance factor for RPT, its impact was relatively inconsequential in the regions where a proportion of PC cells is higher. When comparing the cell survival probability in [^177^Lu]Lu- and [^225^Ac]Ac-PSMA, [^225^Ac]Ac-PSMA exhibited an overall lower probability of cell survival across all hypoxia score ranges in the tumor cell-abundant regions. While both ^177^Lu and ^225^Ac exhibited variable patterns of association with cell survival in three tissues, ^225^Ac had a relatively consistent cell survival compared to ^177^Lu, irrespective of the degree of hypoxia.

In additional analyses, the association between cell survival with hypoxia and PSMA density was analyzed in tumor cell-abundant regions of PC1 tissue using various thresholds (**Figure S4A**). Notably, ^225^Ac exhibited an increased probability of cell death in regions characterized by low PSMA density and high levels of hypoxia, compared to ^177^Lu (**Figure S4B-S4F**).

### *In silico* modeling for other targets: FAP and GRPR

The proposed method is readily adaptable to a diverse spectrum of RPT target molecules and radionuclides, providing a more flexible approach to predict and comprehend the efficacy of RPT. In this study, we extended the application of our model to investigate the spatial distribution and biological effects of GRPR and FAP-targeted RPTs. Although the parameters for PBPK and CRD models vary depending on the characteristics of the ligand in practice, here we assumed that RPTs targeting GRPR and FAP have the similar pharmacokinetic characteristics as those targeting PSMA.

**Figure 6:**
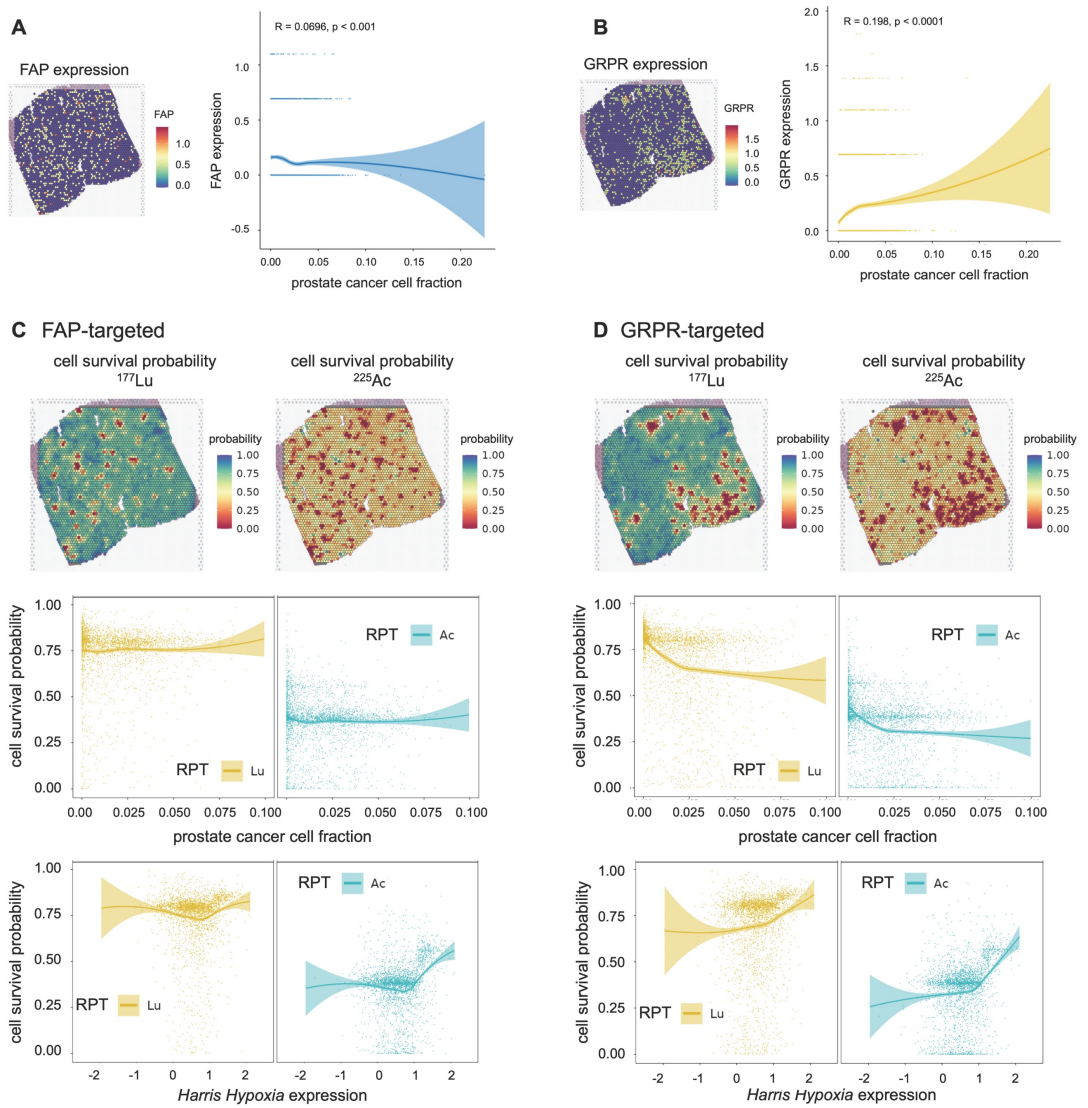
** Cell survival probability of RPTs for two additional targets: fibroblast activation protein (FAP) and gastrin-releasing peptide receptor (GRPR). (A)** The spatial distribution of FAP expression mapped from spatial transcriptomics (ST) of PC1 and the correlation between the prostate cancer cell fraction and FAP expression level. **(B)** The spatial distribution of GRPR expression mapped from ST of PC1 and the correlation between the prostate cancer cell fraction and GRPR expression level. **(C)** The spatial distribution of cell survival probability in [^177^Lu]Lu-labelled FAP-targeted RPT (left, top) and [^225^Ac]Ac-labelled FAP-targeted RPT (right, top). Correlation between cell survival probability and the prostate cancer cell fraction (middle)/hypoxia(bottom) in FAP-targeted RPT. **(D)** The spatial distribution of cell survival probability in [^177^Lu]Lu-labelled GRPR-targeted RPT (left, top) and [^225^Ac]Ac-labelled GRPR-targeted RPT (right, top). Correlation between cell survival probability and the prostate cancer cell fraction (middle)/hypoxia(bottom) in GRPR-targeted RPT. The solid line represents the estimation based on locally estimated scatterplot smoothing (LOESS) local regression fitting, and the area around the solid line indicates the 95% confidence of the fitting lines.

FAP exhibited an overall weakly negative correlation with the tumor cell fraction (Spearman ρ = -0.070, p=0.0001) (**Figure 6A**). The cell survival probability showed a weak correlation with tumor cell fraction (^177^Lu: Spearman ρ = -0.081, p<0.0001, ^225^Ac: Spearman ρ = -0.083, p<0.0001) and mildly increased in the tumor cell-abundant regions for both ^177^Lu- and ^225^Ac-labeled RPTs (**Figure 6C**). Despite this, overall cell survival was lower in ^225^Ac- compared to ^177^Lu-labeled RPTs. In the case of GRPR, the distribution of tumor cells was weakly and positively correlated with the GRPR expression (Spearman ρ = 0.198, p<0.0001) (**Figure 6B**). Accordingly, the tumor cell fraction showed a negative correlation with cell survival probability (^177^Lu: Spearman ρ = -0.408, p<0.0001, ^225^Ac: Spearman ρ = -0.339, p<0.0001) (**Figure 6D**). The cell survival rate was consistently lower in ^255^Ac-labeled RPTs compared to ^177^Lu-labeled RPTs. In both FAP and GRPR, there was a trend toward an abrupt increase in cell survival probability in highly hypoxic regions, which was more pronounced in ^255^Ac-labeled RPTs.

### FAP, GRPR and PSMA

**Figure 7:**
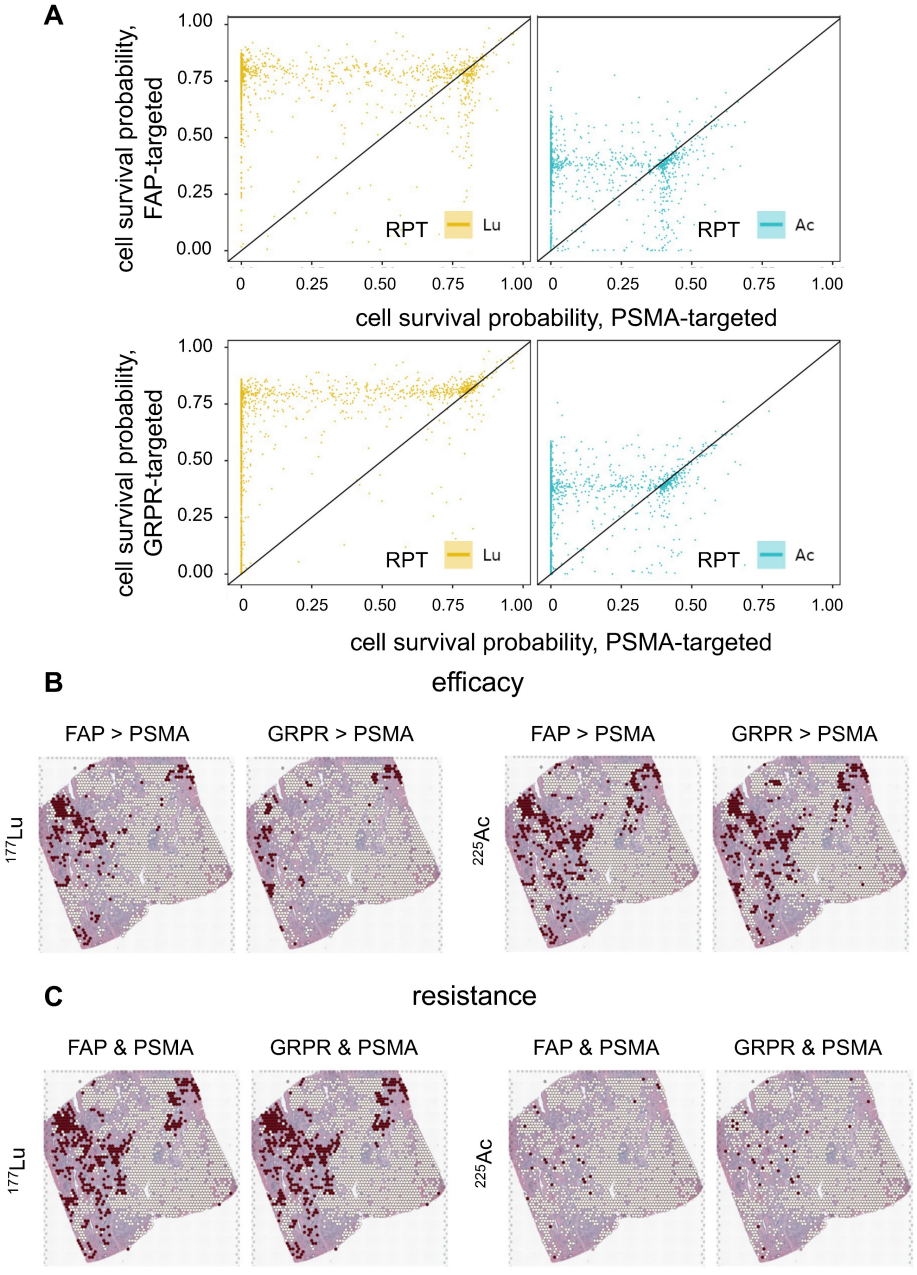
** Efficacy of RPT utilizing various targets: Fibroblast activation protein (FAP), gastrin-releasing peptide receptor (GRPR) and prostate-specific membrane antigen (PSMA). (A)** The scatter plots show the comparison in cell survival probability between FAP-/GRPR-targeted RPT and PSMA-targeted RPT. The dots located below the diagonal line indicate the region where the efficacy of the PSMA-targeted RPT is lower than that of the FAP- or GRPR-targeted RPT. **(B)** The spatial distribution of spots showing higher efficacy in either FAP- or GRPR- compared to PSMA-targeted RPT. **(C)** The spatial distribution of the spots showing resistance in both FAP-PSMA and GRPR-PSMA pairs. The red dots indicate the corresponding locations of the spots, while the white dots represent the remaining spots within the tumor-cell abundant region using threshold of 0.01.

The biological effect of RPTs using different targets, FAP, GRPR, and PSMA, was compared (**Figure 7A**). In general, PSMA exhibited lower cell survival compared to FAP and GRPR in most regions for both ^177^Lu and ^255^Ac-labeled RPTs, indicating the effectiveness of targeting PSMA. Yet, ^225^Ac showed higher treatment efficacy in FAP or GRPR than in PSMA at multiple spots of ST (FAP > PSMA: 11.4%, GRPR > PSMA: 10.25%), while in ^177^Lu, fewer spots showed superior efficacy (FAP > PSMA: 6.8%, GRPR > PSMA: 3.2%) (**Figure 7B**). For FAP, when the corresponding spots were mapped to the tissue, their distribution was concentrated at the tumor cell-depleted region where the PSMA density is lower and FAP density is higher than in the abundant region. In the case of GRPR, fewer spots showed differences with PSMA compared to FAP, and the distinction between FAP and GRPR was less pronounced in ^255^Ac than in ^177^Lu. Also, in ^225^Ac, fewer spots showed ineffectiveness, e.g. cell survival probability above 0.5, in both targets (FAP and PSMA: 0.8%, GRPR and PSMA: 1.0%), compared to those in ^177^Lu (FAP and PSMA: 12.5%, GRPR and PSMA: 13.0%) (**Figure 7C**). When the resistant spots were mapped to the tissue, a substantial overlap was observed with regions identified as more effective in either FAP or GRPR than PSMA, but only for ^177^Lu-labeled RPTs. This suggests that despite limited cell-killing effect with GRPR and FAP in these samples, these two different targets exhibited improved efficacy over PSMA in some regions supporting combination therapy.

## Discussion

RPT has emerged as a promising and safe targeted approach for treating various cancers. In this study, we proposed a novel approach to microscopically analyze the dosimetry and biological effects of RPT by combining novel ST techniques and conventional pharmacokinetic modeling. Utilizing ST data from prostate cancer tissue, we simulated PSMA, FAP, and GRPR-targeted RPTs with both beta- and alpha- emitting ligand accounting for the complex heterogeneities within the TME. In the case of PSMA-targeted RPT, ST spots with a higher PC cell fraction received a higher dose, resulting in lower PC cell survival probability. Even in ST spots with lower PC cell fraction, ^225^Ac demonstrated a lower cancer cell survival probability, indicating its high efficacy in RPT. Our *in silico* model challenged traditional notions, indicating minimal implications of hypoxia resisting PSMA-RPT in both tumor cell-abundant and depleted regions. Yet, [^225^Ac]Ac-PSMA-ligand displayed increased efficacy compared to [^177^Lu]Lu-PSMA-ligand, underscoring its potential in overcoming hypoxic microenvironments. Furthermore, the strong negative correlation of PSMA density with cell survival rate, particularly accentuated in ^225^Ac, adds a layer of intricacy to RPT dynamics, highlighting the importance of considering ligand-specific responses for enhanced therapeutic outcomes. When our proposed *in silico* model was applied to FAP and GRPR, ^225^Ac consistently showed superior efficacy compared to ^177^Lu, although the two targets exhibited distinct spatial patterns of response to RPT. Since FAP had a trend of lower expression at the tumor cell-abundant region than in the depleted region, it showed resistance to RPT in tumor cell-enriched regions. The overall therapeutic efficacy of PSMA was superior compared to FAP and GRPR, assuming that both ligands possess similar pharmacokinetic parameters. These findings emphasize the diverse responses to different targets and radionuclides, underscoring the need for tailored approaches in RPT.

Understanding the biological impact of RPT relies on accurately assessing the absorbed dose, which, in turn, faces challenges due to spatial and temporal heterogeneities^45^. Spatially, RPT deposits nonuniform energy in tissues, influenced by factors such as molecular target distribution and physiological tracer transport^24^,^26^,^27^,^35^. Temporally, the dose rate is determined by the initial activity and half-life, affecting DNA double-strand break induction kinetics and treatment outcome^46^. A recent study showed the heterogeneity of absorbed dose and the biological effect of alpha and beta emitting RPTs by integrating autoradiography with hematoxylin and eosin stained slide from a mouse model of prostate cancer^47^. In the current work, we specifically addressed the spatial complexities of the TME in dosimetry. Leveraging ST, a cutting-edge technique offering genome-wide expression profiling at a micrometer scale, our study pioneers the application of ST to microscopic dosimetry for RPT. This approach comprehensively considers variables such as spatial distribution of the target, diverse cell types, and the functional characteristics of the TME simultaneously.

On a microscopic level, the TME, comprising elements such as blood vessels and CAF, plays a role in altering RPT resistance and clinical outcomes^48^. The targeted dose for this study was 10 Gy 14 days post-injection using the prostate tumor model^29^, and the dose delivered to PC cells was better conformed to the target dose in [^177^Lu]Lu- compared to [^225^Ac]Ac-PSMA-targeted RPT **(Table S1, Figure S2C)**. Yet, in ^225^Ac, the dose distribution was less heterogeneous and more selective to tumor cell-abundant areas. In RPT, factors such as injected activity, vessel distribution, and input function (IF) add complexity to achieving targeted dose precisely. In our investigation, the IF of ^225^Ac was scaled down by a factor of 100 compared to ^177^Lu, aligning with clinical practice of injected dose. Nevertheless, this resulted in lower conformity index (CI) values, particularly in the case of ^225^Ac, suggesting the need for a reevaluation of current injected dose practices in the clinical setting. In particular, the CI values in PC2 were significantly low in both ^177^Lu and ^225^Ac, potentially attributed to the low PSMA density in the tissue and its minimal overlap with vascular matrices **(Figure S2B-S2C)**. Despite both PC1 and PC3 exhibiting high PSMA density, CI values were higher in PC1, indicating better conformity with the targeted dose. Despite low vascularity in PC1, its relatively high overlap with PSMA density may contribute to higher CI values. This underscores the importance of a careful investigation into the current norm of fixed injected dose, considering both PSMA density and its overlap with the vascular component in TME for precise therapy when utilizing PSMA-ligands. Furthermore, the shorter-pathlength beta emitter such as ^177^Lu and alpha emitter such as ^255^Ac travels relatively short range, therefore, their microscale dosimetry in tissue particularly crucial^45^. On the other hand, due to its shorter range and smaller S-value kernel size compared to ^177^Lu, ^225^Ac exhibited a narrower dose distribution with a higher peak **(Figure 3)**, translating into smaller HI and GI values.

Overall, a consistently lower cancer cell survival probability was observed in ^225^Ac compared to ^177^Lu in all three PC tissues **(Figure 4)**. This discrepancy can be attributed to the distinct characteristics of alpha particles, which possess significantly greater energy per disintegration and exhibit a relatively limited penetration range compared to beta particles, categorizing them as high-linear energy transfer (LET) radiation sources^14^. Recent clinical investigations have showcased the efficacy of alpha-emitting radiopharmaceuticals as a salvage therapy subsequent to the inadequacy of beta-emitting RPT^49^-^51^. Although ^177^Lu-labeled RPTs showed a higher average absorbed dose than ^255^Ac-labeled RPTs in the three tissues (**Figure 3**), probably due to the cross-fire effect of ^177^Lu, higher DSB load in ^225^Ac resulted in an overall lower average cancer cell survival. The cell survival probability distributions for both ^225^Ac and ^177^Lu revealed two distinctive peaks, with the second peak in the distribution of ^177^Lu indicating considerable resistance to therapy. The emergence of these dual peaks in the cell survival probability distribution could likely result from the interplay between the heterogeneous target molecule distribution and distance from the vessels **(Figure 2 and Figure 4)**. PC2 and PC3 exhibited greater resistance in both [^177^Lu]Lu- and [^255^Ac]Ac-PSMA-targeted RPT, represented by a higher proportion of spots belonging to the second peak than PC1. This could be attributed to a significant decrease of PSMA density in the tumor cell-abundant and endothelial cell-depleted regions of PC2 and PC3 compared to PC1, leading to inefficient delivery of the RPTs to those areas (**Figure S2D**).

Recent efforts have been directed towards elucidating the factors that influence RPT, encompassing target molecule density, hypoxia, vascularization, and DNA repair pathways^52^. We evaluated the relationship between cell survival probability and RPT influencing factors in the tumor cell-abundant regions, such as target molecule density and hypoxia. Notably, target density displayed a negative correlation with the cell survival rate in PSMA, FAP, and GRPR (**Figure 6C-6D, Figure S5**). In contrast to ^177^Lu, ^225^Ac exhibited a low cancer cell survival probability even in areas with low target density, which can be attributed to the high DSB load induced by alpha particles. However, within ST regions characterized by elevated target density, the cell survival probability did not necessarily exhibit a decreasing trend; in some instances, particularly for PSMA and GRPR, it even displayed a slight increase. In both the ^177^Lu- and ^225^Ac-PSMA targeting RPT graphs, there is a slight trend towards increased cell survival at the very tail of the distributions for PC1 and PC2 **(Figure 5A, Figure S4)**. For GRPR, the spatial expression pattern is sparse with many identical values due to the intrinsic limitation of spatial transcriptomics **(Figure S5B)**. To better represent this, we created a bar graph displaying the median cell survival values within each bin of target expression (e.g., GRPR or FAP), divided into 10 intervals **(Figure S8)**. Notably, a slight increase in cell survival probability was observed in highly target-rich areas, particularly with ^177^Lu- -GRPR targeting RPT, although this effect was confined to just a few spots on the graph's right side. Meanwhile, when RPT is directed at cells in the surrounding TME instead of directly targeting cancer cells, such as through FAP targeting, both ^177^Lu and ^225^Ac may exhibit reduced effectiveness in cancer cell-enriched areas. However, ^225^Ac still maintains a slightly higher efficacy. This assertion has been substantiated through data, confirming its greater effectiveness even in these scenarios. It could explain the intratumoral heterogeneity of biological effect and consistently showed the higher efficacy of ^225^Ac in areas of low target density compared with ^177^Lu-labeled RPT **(Figures 4, Figure 5A)**. In other words, the longer penetration depth in ^177^Lu-labeled RPT leads to a broader crossfire effect in cancer cell rich areas, while we could theoretically show that ^225^Ac proved more efficient in eliminating cells in low target expression areas, because its cancer cell killing relies more on the higher LET.

On the other hand, the simulation revealed no significant correlation between hypoxia and cell survival probability with variable association patterns observed across the three tissues **(Figure 5)**. Tumor hypoxia resulting from inadequate vascular networks can lead to resistance and poor response to RPT^18^. In this study, we used LQ model for ^177^Lu and adapted LQ model for ^225^Ac to account for the significant disparity in DSB rates between the two RPT modalities^29^. When modeling cell survival probability via the LQ model, we considered tumor hypoxia as a critical factor linked to RPT resistance and assigned distinct LQ parameters for each normoxia/hypoxia ST region. In regions characterized by notably intense hypoxia, ^225^Ac exhibited no significant increase of cell survival whereas ^177^Lu showed variable patterns of cell survival change. This partly supports the notion that ^225^Ac, which mostly induces DSB, is less affected by oxidative damage-related SSB than ^177^Lu. The proposed method was further applied to *in silico* modeling of FAP- and GRPR-targeted RPTs. Of note, there was a more abrupt increase of RPT resistance in the highly hypoxic region for both ^255^Ac-labeled FAP and GRPR-targeted RPTs compared to ^177^Lu-labeled RPTs. This may be due to applying the same parameters of the LQ models in the highly hypoxic region as those used for PSMA, which may be suboptimal for other targets such as FAP and GRPR, given their more sparse distribution patterns.

The current *in silico* modeling approach allows for the simulation of biological effect of various RPTs with different targets and isotopes. As ST technology becomes more popular and datasets grow, our method facilitates the screening of RPT efficacy for various types of tumor tissues that show large intertumoral heterogeneities. Besides, as for the comparison between PSMA, FAP, and GRPR, we could further dissect the characteristics of TME by explaining on the microscale efficacy of RPTs where some targets are better than the other. To further refine the suggested model, an in-depth exploration of detailed spatial maps encompassing genes associated with DNA repair pathways is essential. Integrating these maps into both dose and bioeffect simulations will be crucial to finely dissect the influencing factors of RPTs. This approach will enable a more refined stratification of these factors and contribute to a deeper understanding of the therapy's dynamics. Additionally, our method allows for virtual experiments with RPT, accounting for various kinetic parameters and TME heterogeneities, as demonstrated using pharmacokinetic parameters from newly developed ligands 53,54 **(Figure S9, *Supplementary material*)**.

Notwithstanding the significant findings, this study possesses certain limitations that warrant consideration. One of the limitations of this study stems from its sole dependence on ST simulations, which hinders the ability to establish direct causal links behind the variations observed in cell survival probability. While the proposed method offers a valuable opportunity to explore dosimetry and its bioeffects within the tumor microenvironment—a critical area of study—this approach still requires preclinical or clinical validation. Future investigations should prioritize the examination of specific immune or radiation damage biomarkers in tissue samples and compare these findings with the biological effect predictions generated by our proposed model. Additionally, future studies should explore the spatial transcriptomics of samples collected both before and after radiopharmaceutical therapy (RPT) to gain insights into how RPT alters the tumor microenvironment. The sample size in the current study is also limited for drawing broad conclusions. However, despite this limitation, the spatial patterns of biological effects observed from PSMA-targeted radiopharmaceutical therapy across three distinct prostate cancer tissues (PC1, PC2, and PC3) were consistent with prostate cancer cell fractions as depicted in **Figure S3B**. Moreover, GRPR, which is predominantly expressed in the tumor core, exhibited correlation patterns similar to those of PSMA. In contrast, FAP, which is more prominently localized at the tumor periphery, displayed distinct patterns, as shown in **Figure 6**. These findings indicate that our simulations reliably capture the expected spatial distribution and associated biological effects of RPT. Additionally, ST relies on transcript data, which does not directly mirror protein expression. This discrepancy could lead to differences in real-world simulations, despite RNA transcripts typically being indicative of protein expression. Moreover, the simulation process itself is not without challenges. A concern is linked to the inherent limitations of ST, which provides transcriptomic profiles at a resolution spanning dozens of cells rather than at the single-cell level. Considering that ST spots within the tumor cell-abundant region comprise a mixture of tumor cells and stromal cells, the assumption of homogeneity in PSMA density and the degree of hypoxia within each spot may not be completely accurate. Furthermore, although the predicted cell death fraction within the tumor cell-abundant region is primarily associated with the PC cells, it can also be attributed to stromal cells, especially in the case of ^177^Lu, where the crossfire effect is more pronounced. A third complexity lies in the temporal heterogeneity, which was considerably simplified in our simulations. Lethal events associated with dual DNA DSBs manifest when the time interval between two distinct radiation incidents results in the misrepair of two SSBs during the repair process. If the time interval between the first and second DSBs is substantial, the first DSB may undergo repair prior to being improperly joined with the second DSB. This intricate process, elucidating the impact of dose rate and fractionation on the yield of lethal damage and ensuing cell survival, was simplified via the utilization of the LQ model, with parameter values adopted from prior literature. It's important to note that we did not explore the effects of dose rate and cell cycle in this study.

## Methods

### Generation of microscopic scale tumor microenvironment (TME) maps using ST

Three publicly available Visium ST datasets were collected from primary tissue samples of two prostate cancer patients: one diagnosed with acinar cell carcinoma (PC1: https://www.10xgenomics.com/resources/datasets) and the other with adenocarcinoma (PC2 and PC3: https://data.mendeley.com/datasets/svw96g68dv/1). Subsequently, the obtained data were processed by applying the normalization and log-transformation (Seurat package, V4.1.1)^55^.

Various gene expressions (*FOLH1* and Harris Hypoxia) were extracted in each ST spot locations and the proportions of cell type (epithelial, endothelial, and PC cell) were estimated in corresponding ST spots. The resulting maps were used to delineate the vessel (endothelial cell), hypoxia (*Harris Hypoxia*), PSMA binding density (*FOLH1*), and PC cell regions for the dosimetry and their biological effect simulation in this work. *FOLH1* is the gene that encodes PSMA, and Harris Hypoxia is a gene set related to hypoxia 39. The enrichment scores of gene expressions were calculated (GSVA package, V1.48.1)^56^,^57^, and the single-sample Gene Set Enrichment Analysis (ssGSEA) method was applied with Poisson kernel to the raw count data. On the other hand, the proportion of each cell type in given ST spot was estimated by employing two public scRNA-seq datasets from PC patients^58^,^59^.

In particular, the cell type deconvolution method, CellDART^44^ were utilized. CellDART first trains the model to predict cell composition from virtual cell mixtures created from the scRNA-seq dataset and then adapts the neural network to predict the composition from real ST spots. During the analysis, the raw count matrices from both scRNA-seq and ST datasets were used, along with the cell type annotation information provided in the literatures^58^,^59^. All analyses were performed using the default parameters suggested in the user guide.

The disparities in spatial distribution across the three datasets were analysed by measuring the relative volumes, overlap, and dissimilarity between vessel, hypoxia, and PSMA binding density maps. The relative volume was determined as the ratio between the number of ST spots containing the respective binarized maps using k-mean clustering and the total number of ST spots. Additionally, relative PSMA density was defined as the mean value over the maximum value of the PSMA density map. The dissimilarity between two maps was calculated using Kullback-Leibler divergence (KLD).

### Spatiotemporal biodistribution of [^177^Lu]Lu- and [^225^Ac]Ac-PSMA-ligand

Spatiotemporal biodistributions of [^177^Lu]Lu- and [^225^Ac]Ac-PSMA-ligand were simulated on the ST domain using the triangular mesh **(Figure S6A- S6B)**. We used MATLAB's PDE Toolbox with a mesh generated using generateMesh, setting Hmin to 80 µm and Hmax to 160 µm for a balance between accuracy and efficiency, resulting in an approximate mesh edge size of 100 µm **(Figure S6B)**. The radiopharmaceutical initially enters the tumor interstitium from the vasculature through a combination of diffusion along the concentration gradient and convection driven by interstitial fluid pressure (IFP) gradients^29^. Thereafter, it encounters the cellular compartment, which we further divide into two additional compartments: the bound compartment and the internalized compartment. Upon binding to PSMA expressed on the surface of prostate cancer cells, the radiopharmaceutical undergoes an internalization process, resulting in its concentration within the cell. Consequently, the concentration of the radiopharmaceutical in the three compartments—interstitium (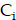), bounded (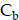), and internalized (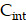)—can be mathematically expressed as follows:


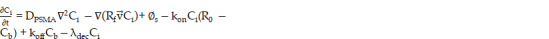
 (Equation 1)


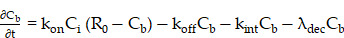
 (Equation 2)


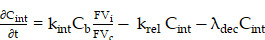
 (Equation 3)

The time derivative of 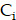 can be described by a CRD model. The diffusion term, 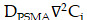, is governed by 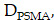 diffusivity of PSMA-ligands. The convection term, 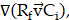 is defined by 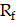, molecule/carrier movement coefficient, and 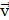, velocity imposed by IFP ***(Supplementary Material).*** The reaction term encompasses the remaining components in **(Equation 1)**. The source term, denoted by 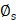, is determined by the input function (IF) of the vessel map ***(Supplementary Material)***. Given that our samples are from prostate tissue, we used the physiologically based pharmacokinetic (PBPK) model to estimate the time-activity curve (TAC) of IF within the prostate's vasculature 29,60. The injected amount of IF is carefully selected to deliver a mean absorbed dose of 10 Gy to the prostate after 14 days post-injection by PBPK model **(Figure S6C)**, considering the clinical relevance 13. The vasculature contour was delineated by applying a threshold to the vessel map, with the cut-off established as the mean value between two cluster centers defined through k-means clustering. The association and dissociation rates are represented by 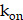 and 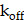, respectively. The receptor density, 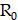, is proportionally adjusted by the PSMA binding density map, and 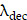 denotes the decay constant specific to each radiopharmaceutical. Specifically, the PSMA binding density map underwent binarization, employing a threshold derived similarly to the vasculature contour. Subsequently, the PSMA binding density map was normalized by its maximum value, and the resulting map was utilized as a scaling factor for 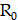.

The rates of change of 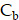 and 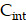 are modeled using first-order kinetics. The internalization rate is represented by 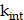, while the release rate is denoted by 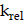. The fractional volumes of tumor interstitium and cells within each ST spot are represented by 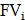 and 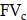, respectively.

To solve the system of PDE **(Equation 1-3)**, we employed the MATLAB PDE Toolbox (MathWorks, Natick MA). Neumann boundary conditions were applied, specifying a normal derivative at the vessel edge to be zero **(Figure S6B)**. The concentration 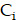, 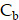 and 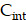 were solved at every time step, with calculations performed in five-minute intervals over a period of 14 days. The parameters used in the simulations were obtained from previous the literature **(Table S2-S3)** 29,61. The simulation was repeated 20 times for each tissue sample and for both ^177^Lu and ^225^Ac-labelled RPT, and the reported results were derived from the average of these simulations. The coefficient of variation (CV) was measured to provide the robustness of the simulation.

### Calculation of absorbed dose

According to the MIRD formalism^20^,^62^,^63^, the mean absorbed dose D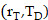 to target tissue 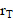 over a defined dose-integration period (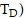
after administration of the radioactive material to the subject is given as:


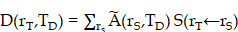
 (Equation 4)

where 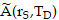
is the time-integrated activity (TIA), calculated by 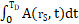 , in source tissue 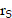 over the period 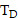. In this work, we numerically integrated 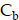 and 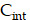
over a duration of 14 days to obtain the TIAs, considering C_i_ was negligible **(Figure S6C)**. The quantity 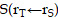 is specific to the radionuclide and to the computational phantom defining the spatial relationship and tissue compositions of 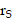 and 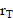 and their intervening tissues in the reference individual or tissue model. To determine S-values for ^177^Lu and ^225^Ac, we utilized the DPK method, and the kernel values were derived from MIRDcell (V2.0) **(Figure S6D)**^21^. MIRDcell is a tool that adapts the MIRD formalism for cellular and subcellular dosimetry. It models the dose to the cellular and subcellular compartments (i.e., the cell membrane, cytoplasm, and nucleus) for both isolated cells and clusters of cells using cellular S values and mathematically models the responses of labeled and unlabeled cells as a function of the fraction of cells labeled.

### Biological effect modeling

The dose-dependent cell survival can be described by the target theory-based linear quadratic (LQ) model. The survival probability, 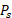, is calculated using the equation:


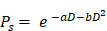
 (Equation 5)

Here, D represents the absorbed dose, 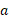 is the linear sensitivity coefficient and 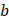 is the quadratic sensitivity coefficient. Cells with a higher *a* and *b* are more sensitive to radiation^64^. For [^225^Ac]Ac-PSMA-targeted RPT, which induces more DNA DSBs, 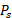 can be modeled as^29^:


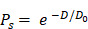
 (Equation 6)

In this equation, 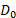 represents the absorbed dose required to yield a survival fraction of 37%.

The LQ model parameters are influenced by tissue oxygenation. Cells in well-oxygenated tissues tend to be more radiosensitive resulting in a more effective killing effect of RPT. Therefore, in our study, we divided the regions into normoxia (oxygen-abundant) and hypoxia (oxygen-depleted) based on the hypoxia map by setting a threshold at the mean value of third and fourth cluster center defined through k-means clustering of the hypoxia map. Different sets of *a*,* b,* and 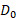
values were assigned to each region **(Table S4)**.

### Stability of the simulation

To address the stability of PDE solution, simulations were conducted 20 times for each PC1, PC2, and PC3 sample for PSMA-targeted RPTs, and the coefficient of variation (CV) for dose distribution and cell survival probability was calculated for each spot location in the tissues (**Figure S7**). CVs were spatially mapped to the tissue location, and the association between CV and various parameters was investigated, including endothelial cells, prostate cancer cells, *FOLH1* expression, and *Harris Hypoxia* scores. The CV of absorbed dose increased in regions with high *FOLH1* expression and a higher proportion of prostate cancer cells in PC1. However, this trend was not as clear in PC2 and PC3 tissues. On the other hand, the negative association between CV of cell survival probability and *FOLH1* expression, as well as prostate cancer cell fraction, was more consistent and prominent.

### Dose analysis using dose-volume histogram (DVH)

The efficacy of RPT relies on its ability to deliver cytotoxic radiation specifically to cancer cells or the surrounding TME while minimizing normal tissue toxicity. Thresholds (0.005, 0.01, 0.02, and 0.05) in the PC cell map were varied to investigate alterations in dose distribution and treatment efficacy based on the proportion of cancer cells within each spot. For the study, the tumor cell-abundant/-depleted regions were designated by applying a cut-off of 0.01, unless mentioned otherwise. DVH analysis provides insight into the radiation dosage received by specific tissue volumes, allowing for a comprehensive evaluation of treatment outcomes. RPT efficacy was assessed by plotting the DVH within each tumor cell-abundant/-depleted/entire region. Subsequently, various indices including conformity indices (CI), homogeneity indices (HI), and gradient indices (GI) were analyzed based on the DVH, commonly used in evaluating dose distribution in external beam radiotherapy (EBRT) **(Table S1)**. The CI assesses the agreement between the irradiated volume and the target volume, while the HI evaluates the uniformity of dose distribution within the target volume. Furthermore, the GI indicates information about the steepness of the dose falloff outside the target volume 65. The target volume, identified as the area abundant in tumor cells, was determined by applying a threshold of 0.01 to the PC cell map. The target dose was 10 Gy, and we conducted a subsequent measurement of all indices, adjusting the dose distribution to center the average around the target dose through mean normalization.

### Application to FAP and GRPR

Given the extensive scope of clinical trials in RPT, the methodology was extended to assess dose and its bioeffect in FAP- and GRPR-targeting RPT. It was assumed that the radiopharmaceuticals employed in both RPT scenarios exhibit similar kinetics to PSMA-ligand. The FAP and GRPR maps were extracted and processed using identical procedures. In summary, this method can be used to investigate the intratumoral heterogeneity of biological effects for RPTs in targets other than PSMA.

### Statistical analysis

Pearson's correlation was assessed to examine the correlation between the ST maps of various gene expressions and cell types. To compare the dose distribution between [^177^Lu]Lu- and [^225^Ac]Ac-PSMA-ligand within different subregions (tumor cell-abundant/-depleted/-entire region), a paired t-test was conducted. Additionally, a paired t-test was employed to analyze the disparity in cell survival probability between [^177^Lu]Lu- and [^225^Ac]Ac-PSMA-ligand. To assess the potential impact of PSMA density and hypoxia on the efficacy of PSMA-targeted RPT, Spearman's rank correlation was measured which evaluates the monotonic relationship between these influencing factors and each dose and cell survival probability distribution.

## Supplementary Material

Supplementary information, figures and tables.
