# Supplementary material for: Deciphering the effects of radiopharmaceutical therapy in the tumor microenvironment of prostate cancer: an in-silico exploration with spatial transcriptomics
Source: Theranostics. 2024 Oct 28;14(18):7122–39. doi: 10.7150/thno.99516 (PMC11610134; doi:10.7150/thno.99516)
Supplement: Supplementary file 1 — Supplementary information, figures and tables. [file thnov14p7122s1.pdf]

Supplemental Materials

Solving the convection term in CRD

The convection term in the solving PDE for the concentration of radioligand in interstitial compartment ($\boldsymbol{C_i}$) is expressed as:

$$\nabla(\mathrm{R_f}\vec{\mathrm{v}}\mathrm{C_i}) = \mathrm{R_f}\nabla(\vec{\mathrm{v}}\mathrm{C_i}) = \mathrm{R_f}((\nabla\mathrm{C_i})\cdot\vec{\mathrm{v}} + \mathrm{C_i}\,(\nabla\cdot\vec{\mathrm{v}})) \qquad \text{Equation 1}$$

where $\mathbf{R_f}$ is defined as molecule/carrier movement coefficient.

Tumor capillaries are leakier than normal capillaries, hence, liquid carrying the tracer can easily enter the tumour extracellular compartment, influencing the tracer distribution within the tumour interstitium compartment[58,59]. The flow velocity in the tumour extracellular matrix, $\vec{\boldsymbol{v}}$ can be modelled using Darcy's law which describes low speed liquid flow in porous media as:

$$\vec{v} = -\,K_p\nabla p_i \qquad \text{Equation 2}$$

where $\boldsymbol{p_i}$ is the interstitial fluid pressure (IFP) in the tumour interstitium and $\boldsymbol{K_p}$ is the hydraulic conductivity.

By the law of mass conversation,

$$\nabla\cdot\vec{v} = -\,K_p\nabla^2 p_i = \varphi_v(x,y) \qquad \text{Equation 3}$$

Here, $\boldsymbol{\varphi_v(x,y)}$ is a source term describing liquid leakage into the tumour interstitium through the capillary wall, and according to Starling's law[60] we can model the source term as:

$$\varphi_v(x,y) = [(p_v - p_i) - \sigma_T(\pi_v - \pi_i)]\cdot L_p\cdot\left(\frac{S_b}{V_i}\right)_{avg}\cdot\left(\frac{N_{tot}}{N_{blood}}\right)\cdot B(x,y) \qquad \text{Equation 4}$$

where $\boldsymbol{p_v}$ is the capillary hydrostatic pressure; $\boldsymbol{\sigma_T}$ is the reflection coefficient; $\boldsymbol{\pi_v}$ and $\boldsymbol{\pi_i}$ represent capillary and tumour interstitial oncotic pressures; $\boldsymbol{L_p}$ is the hydraulic permeability of the microvascular wall; $\left(\frac{\boldsymbol{S_b}}{\boldsymbol{V_i}}\right)_{\boldsymbol{avg}}$ is the average ratio of capillary surface area to interstitial volume; $\boldsymbol{N_{tot}}$ and $\boldsymbol{N_{blood}}$ are the total number of matrix elements and the number containing blood vessels; and the binary map $\boldsymbol{B(x,y)}$ is derived from the vessel map by applying a threshold. The cut-off for this threshold is determined as the mean value between two cluster centers, which are defined through k-means clustering. Different sets of parameters were assigned to each (x,y) location depending on whether or not the spot includes tumor cell.

The IFP map was first determined, by solving p_i described **(Equation 3-4)**. The derived IFP was then used to derive the convection term by substituting each $\vec{\boldsymbol{v}}$ and $\boldsymbol{\nabla\cdot\vec{v}}$ term **(Equation 2-3).** the parameter used for solving $\boldsymbol{p_i}$ is summarized **(Table S2)**, and the solved IFP map was illustrated (**Figure S6A)**.

Source term, $\emptyset_s$

In order to define the sets of PDEs, the source term, $\emptyset_s$, was defined as the flux entering the interstitial compartment from the vessel compartment. In this work, $\emptyset_s$ was computed as:

$$\emptyset_s = (C_v - C_i)\cdot L_v\cdot\left(\frac{S_b}{V_i}\right)_{avg}\cdot\left(\frac{N_{tot}}{N_{blood}}\right)\cdot B(x,y) \qquad \text{Equation 5}$$

Here, $\boldsymbol{C_v}$ is concentration of vessels in prostate tissue, which is given as IF **(Figure S6C)**. Given that our samples are from prostate tissue, we used the PBPK model to estimate the $\boldsymbol{C_v}$ within the prostate's vasculature. $\boldsymbol{L_v}$ represents capillary vessel wall permeability; $\left(\frac{S_b}{V_i}\right)_{avg}$ and $\frac{N_{tot}}{N_{blood}}$ denotes capillary surface area per interstitial volume and total number of matric elements/ number containing blood vessels, respectively. The parameter values used for this work is summarized **(Table S3)**.

Pharmacokinetic parameters for FAP- and GRPR targeting RPT

1. **FAP-targeted RLT (^{177}Lu-DOTAGA-(SA.FAPi)2)[53]**

Given Data (Kd, efflux data) from literature:

- **Dissociation constant ($\boldsymbol{K_\mathrm{d}}$)** 1.35 nM
- **Internalization rate and specific bound uptake after 6 hours**: 14.7%
- **Externalized after 24 hours**: 48% (Fraction Remaining Internalized = 0.52)

2. **GRPR-targeted RLT (^{177}Lu-DOTA-gluBBN)[54]**

Given Data (Kd, efflux data) from literature:

- **Dissociation constant ($\boldsymbol{K_\mathrm{d}}$)** 0.63 nM
- **Internalization rate after 2 hours**: 90.1%
- **Externalized after 2 hours**: 56.3%(Fraction Remaining Internalized = 0.437)

Without actual experimental data for the observed association rate constants at a known ligand concentration during the binding experiments. To calculate $\boldsymbol{k}_\mathrm{on}$, $\boldsymbol{k}_\mathrm{off}$, $\boldsymbol{k}_\mathrm{int}$, $\boldsymbol{k}_\mathrm{rel}$ using the data from the literature (1,2), we used the following relationships and approximations.

Assumptions and Approximations:

- Steady-state conditions are assumed for binding and internalization.
- We assume the association and dissociation processes are in equilibrium.
- The internalization and release rates are first-order processes.
- For biological systems, typical values for k_on range from 10^5 to $10^8\ \mathrm{M^{-1}\cdot s^{-1}}$. However, as the literatures (1,2) does not reveal the observed association rate constants, we approximated k_on to be the same as the value in the manuscript (cited [29] in the submitted manuscript).:

$$\boldsymbol{k}_\mathbf{on} = 8 \times 10^5\ \mathrm{M^{-1}\cdot s^{-1}} = 8 \times 10^{-1}\ \mathrm{mL\cdot nmol^{-1}\cdot s^{-1}}$$

a) **Dissociation Rate Constant($\mathbf{k_{off}}$)**:

$$K_d = \frac{k_\mathrm{off}}{k_\mathrm{on}}$$

$$k_\mathrm{off} = K_d \cdot k_\mathrm{on}$$

b) **Internalization Rate Constant($\mathbf{k_{int}}$)**:

Given internalization fraction ($Fr_\mathrm{internalized}$) at the given time (t), use formula:

$$Fr_\mathrm{internalized} = 1 - e^{-k_\mathrm{int}\cdot t}$$

$$k_\mathrm{int} = \frac{-ln(1 - Fr_{internalized})}{t}$$

c) **Release Rate Constant($\mathbf{k_{rel}}$)**:

Given externalization fraction ($Fr_{\text{externalized}}$) at the given time (t), use formula:

$$Fr_{\text{externalized}} = 1 - e^{-k_{\text{rel}} \cdot t}$$

$$k_{\text{rel}} = \frac{-ln(1 - Fr_{externalized})}{t}$$

For ^{177}**Lu-DOTAGA-(SA.FAPi)2**

$$\boldsymbol{k_{off}} \approx 1.08 \times 10^{-3}\ \text{s}^{-1}$$
$$\boldsymbol{k_{int}} \approx 7.60 \times 10^{-6}\ \text{s}^{-1}$$
$$\boldsymbol{k_{rel}} \approx 9.52\ \times 10^{-6}\ \text{s}^{-1}$$

For ^{177}**Lu-DOTA-gluBBN**

$$\boldsymbol{k_{off}} \approx 5.04 \times 10^{-4}\ \text{s}^{-1}$$
$$\boldsymbol{k_{int}} \approx 3.19 \times 10^{-4}\ \text{s}^{-1}$$
$$\boldsymbol{k_{rel}} \approx 8.69\ \times 10^{-5}\ \text{s}^{-1}$$

Supplementary Figures

A

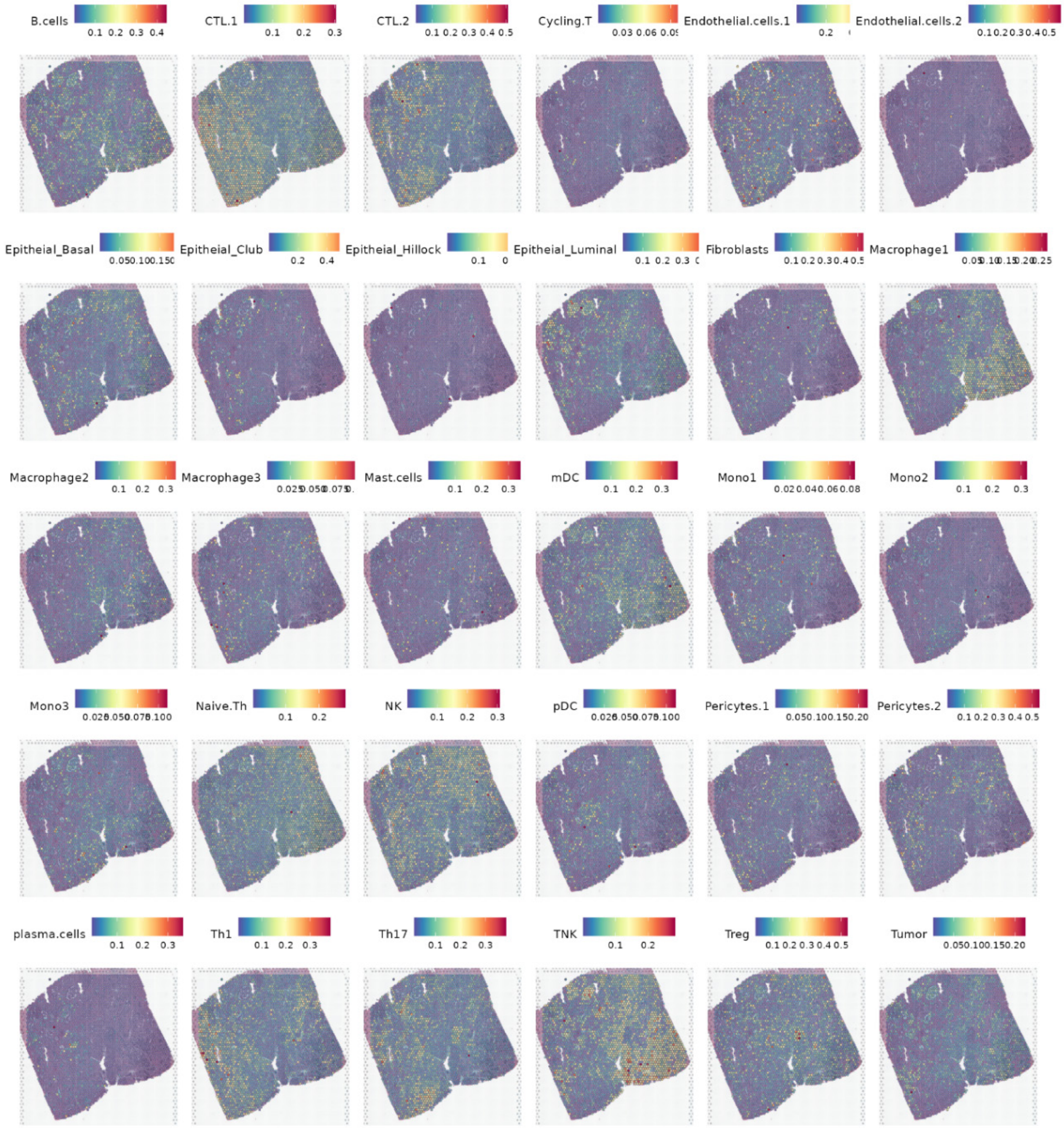

B

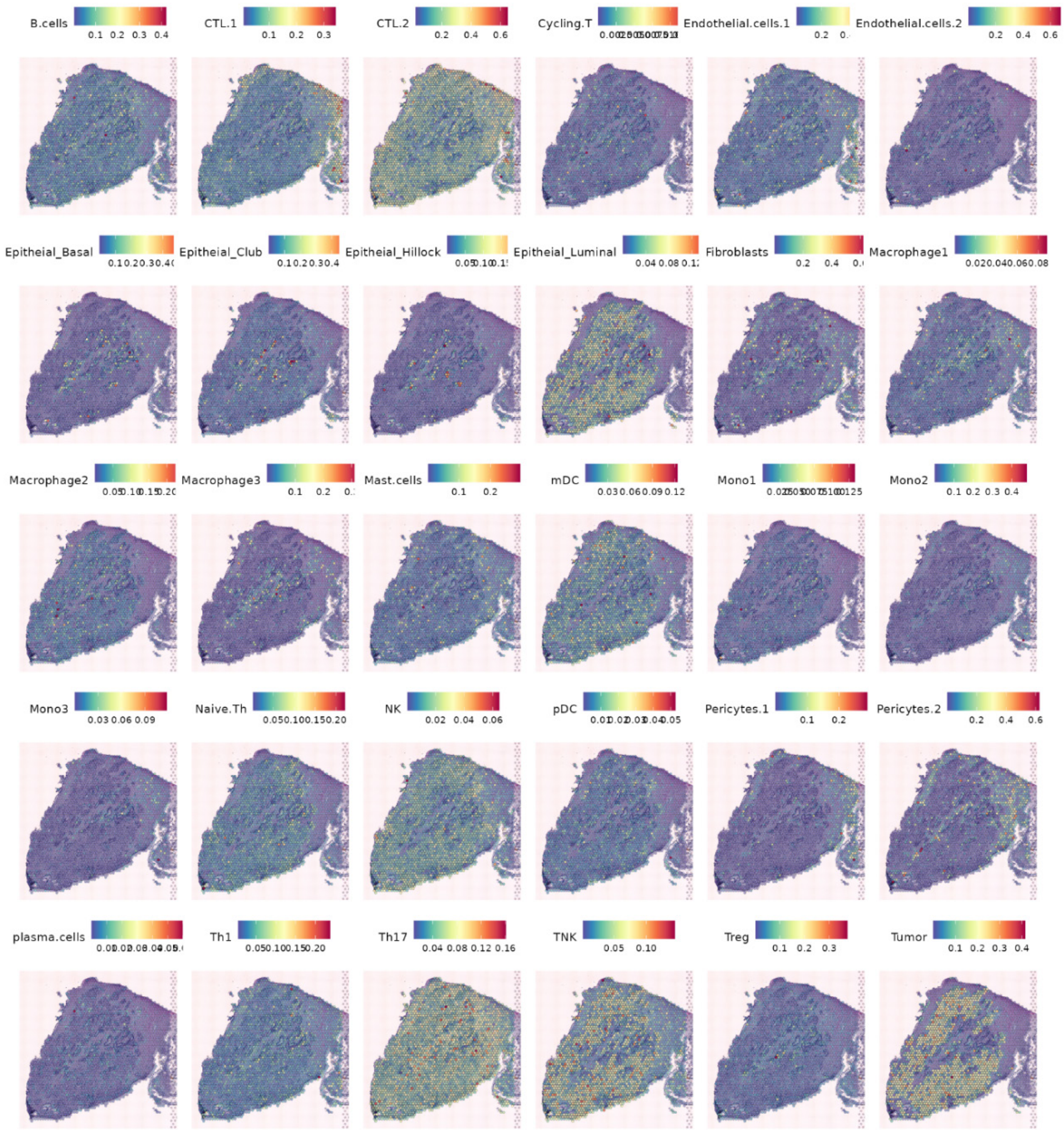

B.cells
CTL.1
CTL.2
Cycling.T
Endothelial.cells.1
Endothelial.cells.2
Epitheial_Basal
Epitheial_Club
Epitheial_Hillock
Epitheial_Luminal
Fibroblasts
Macrophage1
Macrophage2
Macrophage3
Mast.cells
mDC
Mono1
Mono2
Mono3
Naive.Th
NK
pDC
Pericytes.1
Pericytes.2
plasma.cells
Th1
Th17
TNK
Treg
Tumor

C

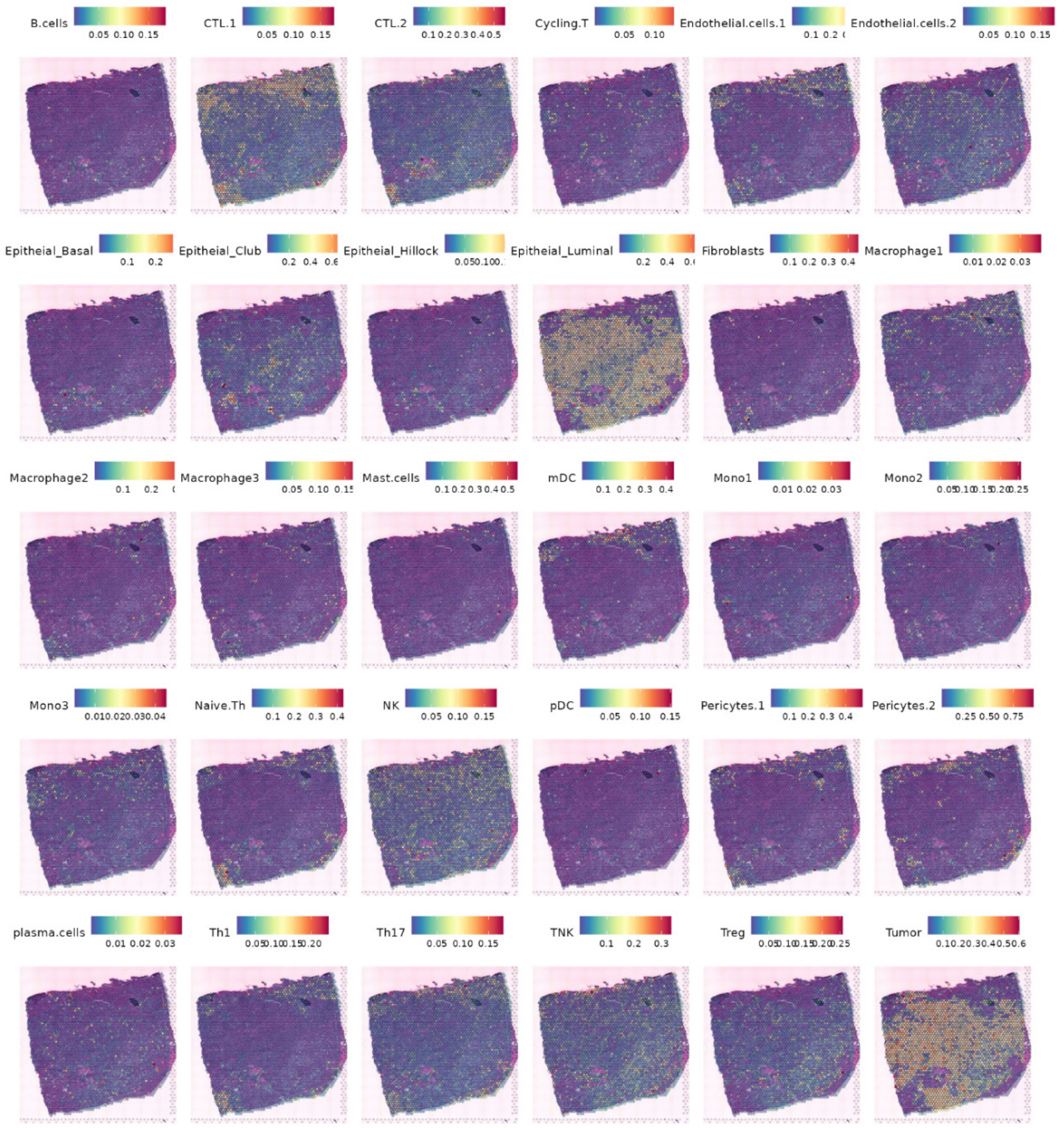

Figure S1. Spatial Transcriptomics (ST) maps of various cell types in the prostate cancer tissues (A) PC1, (B) PC2, and (C) PC3. The color scale for each cell type represents the fraction (probability) of that specific cell type in each ST spot, with all cell type fractions summing up to 1, reflecting the cellular composition in each ST location.

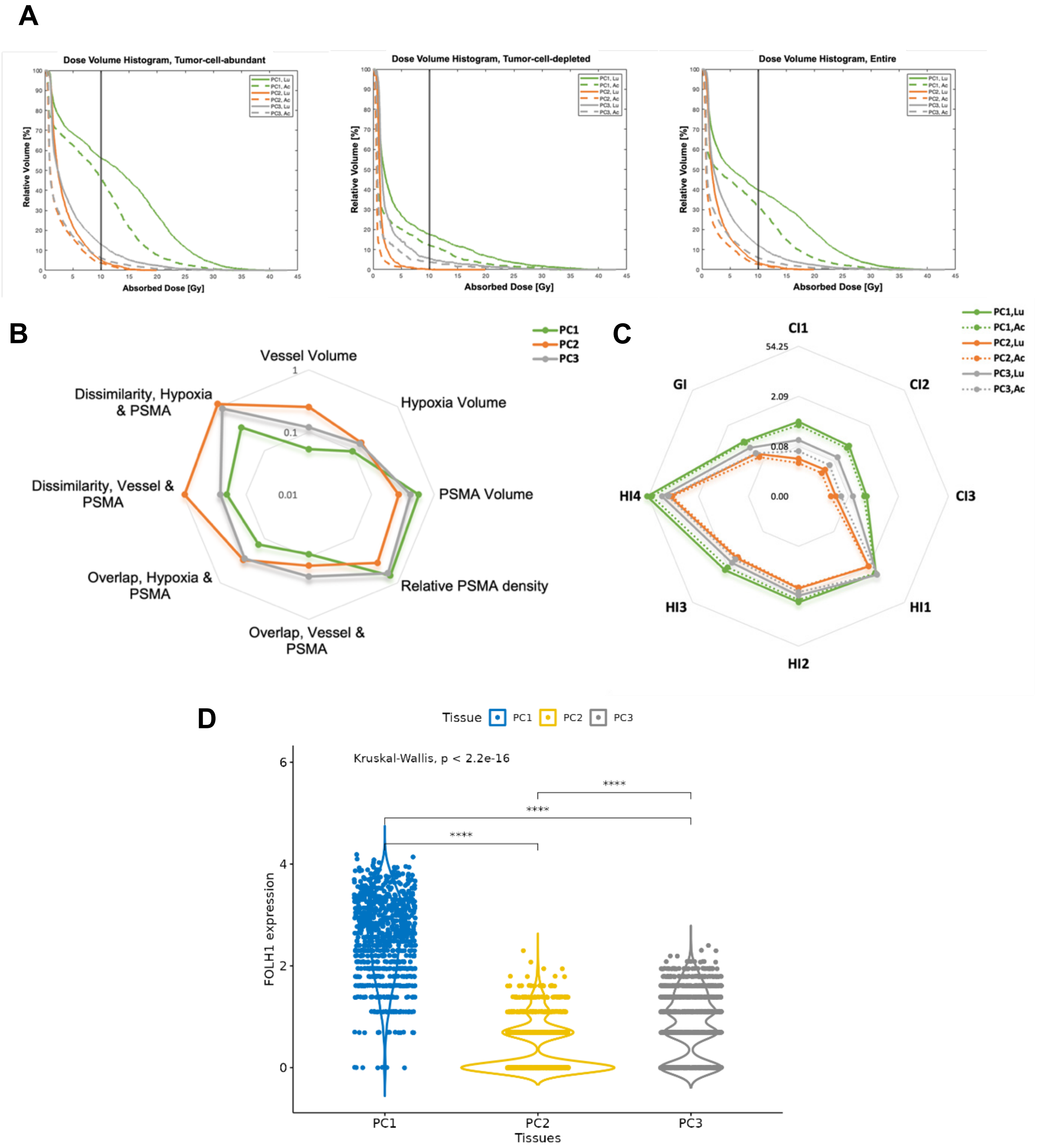

Figure S2. Heterogeneity of spatial distribution and Dose Volume Histogram (DVH) in three prostate cancer tissue sample (PC1-Green; PC2 -Orange; PC3-Gray;). (A) DVH within subregions (tumor cell-abundant/depleted/entire region) is illustrated. DVH illustrates the relationship between the absorbed dose and relative volume affected by corresponding dose. Each DVH within tumor cell-abundant/depleted/entire region is depicted. Solid line and dotted line represent DVH in ^{177}Lu- and ^{225}Ac-PSMA targeting RPT, respectively. The black vertical line indicates the targeted dose. **(B)** The radar chart shows the indices that describe the spatial profiles of PC1, PC2, and PC 3. **(C)** The radar chart illustrates the conformity indices (CI), homogeneity indices (HI), and gradient indices (GI) of ^{177}Lu and ^{225}Ac PSMA-targeted RPT. **(D)** Correlation between endothelial cell proportion and target molecule expression in the cancer tissues. The violin plot illustrates the distribution of the FOLH1 expression at the intersection of endothelial cell-depleted (below the median of the endothelial cell fraction) and cancer cell-abundant (tumor cell fraction above 0.01) regions in PC1, PC2, and PC3 tissues. The Kruskal-Wallis test was employed to demonstrate the statistical difference among the three groups, and pairwise Wilcoxon-tests served as post-hoc examinations. ****: p<=0.0001.

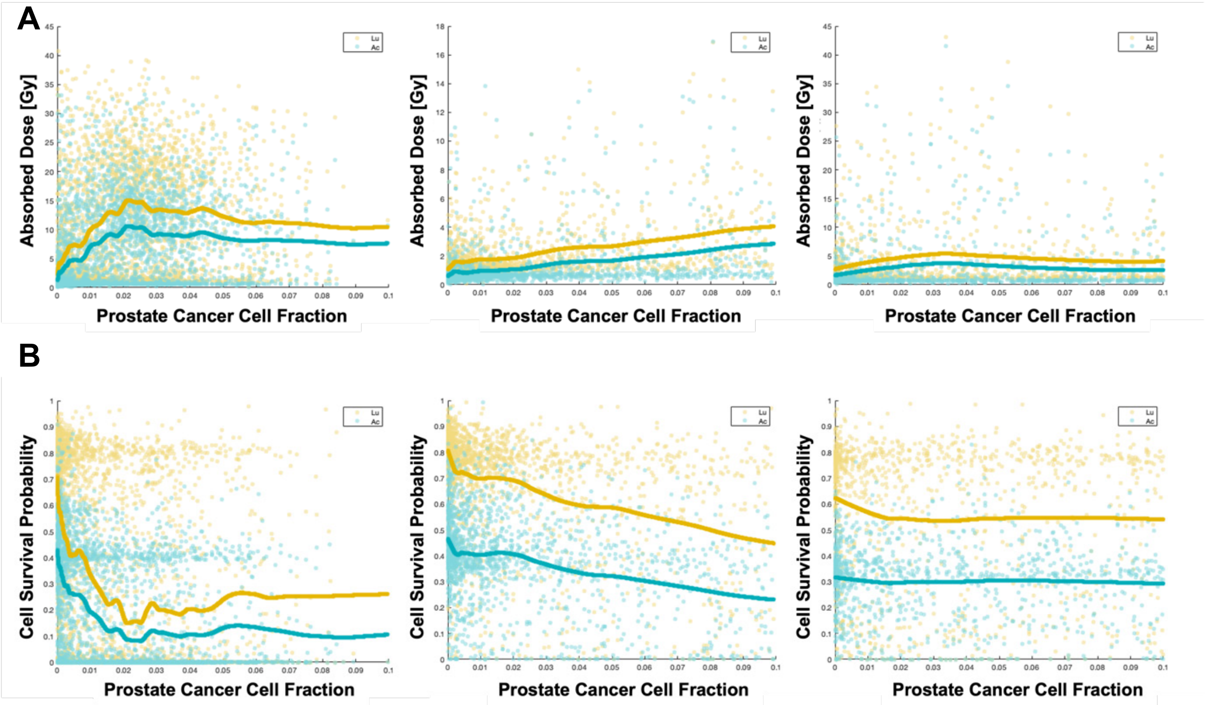

Figure S3. Tumor cell Fraction and the dose/cell survival probability. The distributions of tumor cell fraction and (**A)** absorbed dose (PC1-left, PC2-middle, PC3-right) (**B)** cell survival probability (PC1-left, PC2-middle, PC3-right) in ^{177}Lu (yellow) and ^{225}Ac (turquoise) RPT simulation are illustrated. The solid line indicates the locally weighted smoothing (Loess) curve fitted with each distribution.

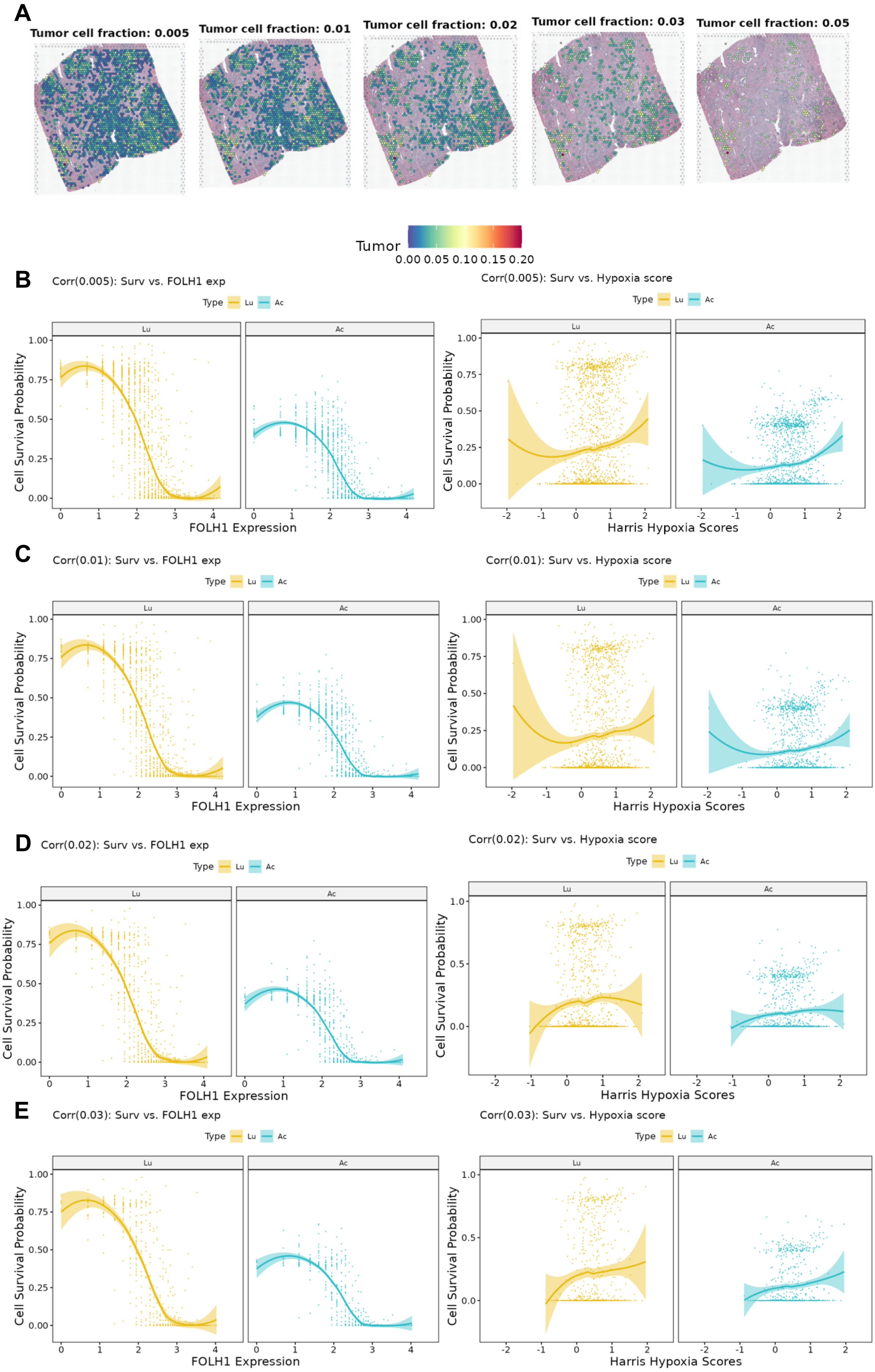
A
Tumor cell fraction: 0.005
Tumor cell fraction: 0.01
Tumor cell fraction: 0.02
Tumor cell fraction: 0.03
Tumor cell fraction: 0.05
Tumor
0.00 0.05 0.10 0.15 0.20
B
Corr(0.005): Surv vs. FOLH1 exp
Corr(0.005): Surv vs. Hypoxia score
C
Corr(0.01): Surv vs. FOLH1 exp
Corr(0.01): Surv vs. Hypoxia score
D
Corr(0.02): Surv vs. FOLH1 exp
Corr(0.02): Surv vs. Hypoxia score
E
Corr(0.03): Surv vs. FOLH1 exp
Corr(0.03): Surv vs. Hypoxia score
Type Lu Ac
Lu
Ac
Cell Survival Probability
FOLH1 Expression
Harris Hypoxia Scores

F

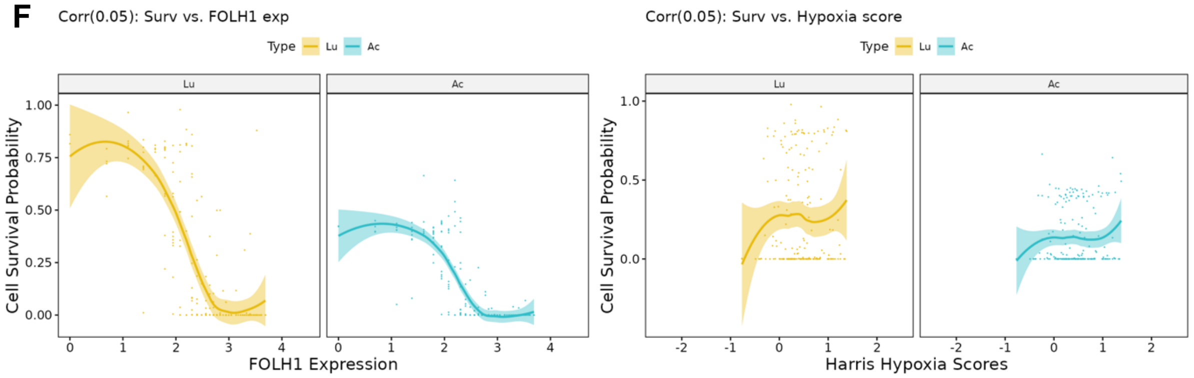

Figure S4. Correlation between influencing factors of PSMA-targeted RPT in Tumor cell-abundant/depleted regions. (A) Tumor cell-abundant/depleted regions. The tumor cell-abundant regions are outlined by applying various threshold to tumor cell fraction: 0.005, 0.01, 0.02, 0.03, and 0.05. Each ST spot covers gene expression within a diameter of 55 micrometers, with a distance of 100 micrometers between the spots. Correlation between influencing factors (PSMA density - left, hypoxia - right) of PSMA-targeted RPT and cell survival probability in the tumor cell-abundant regions, defined by thresholds of tumor fraction **(B)** 0.005, **(C)** 0.01, **(D)** 0.02, **(E)** 0.03, and **(F)** 0.05. The solid line represents the estimation based on Loess local regression fitting, and the area around the solid line indicates the 95% confidence of the fitting lines for the PSMA density and cell survival probability (left) and hypoxia and cell survival probability (right).

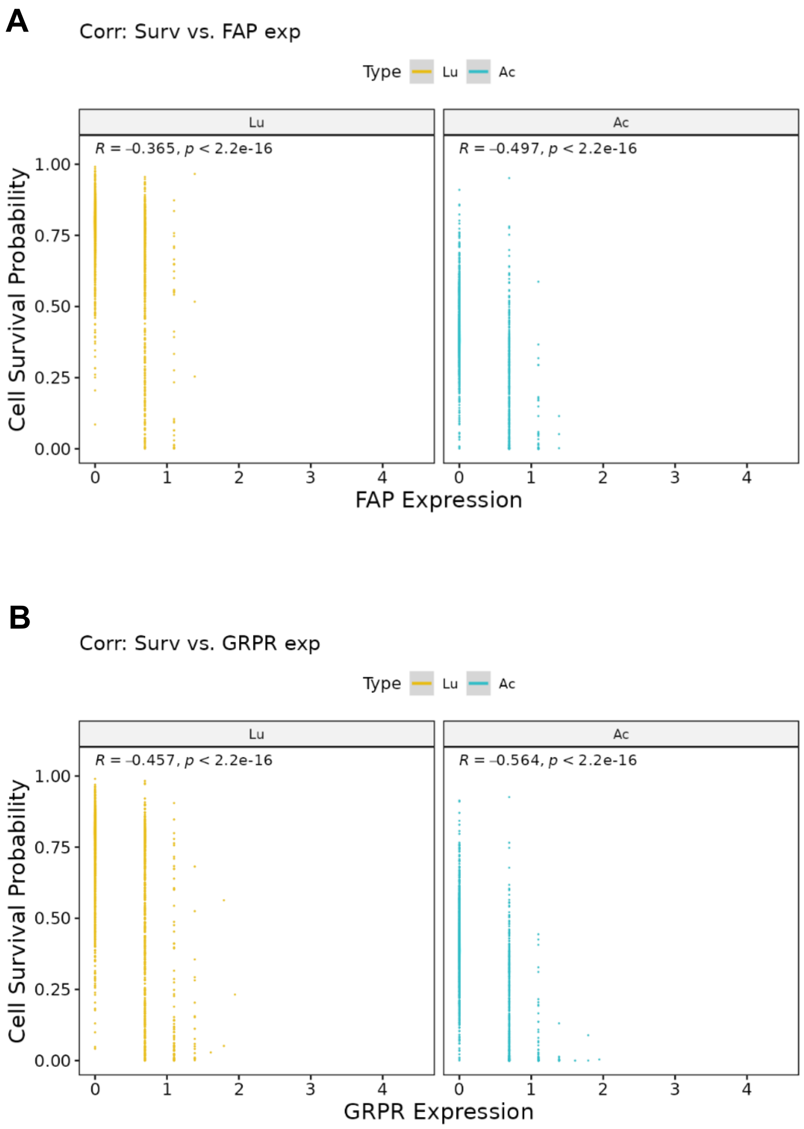

Figure S5. Correlation between the target molecule density and cell survival probability in the PC1 tissue. Spearman's correlation coefficient (R) and statistical significance (p-value) were visualized in the upper left corner of each upper left corner of each plot. **(A)** FAP and **(B)** GRPR density and cell survival.

A

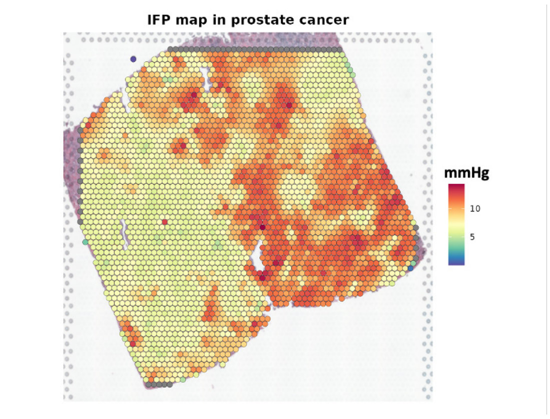

IFP map in prostate cancer
mmHg
10
5

B

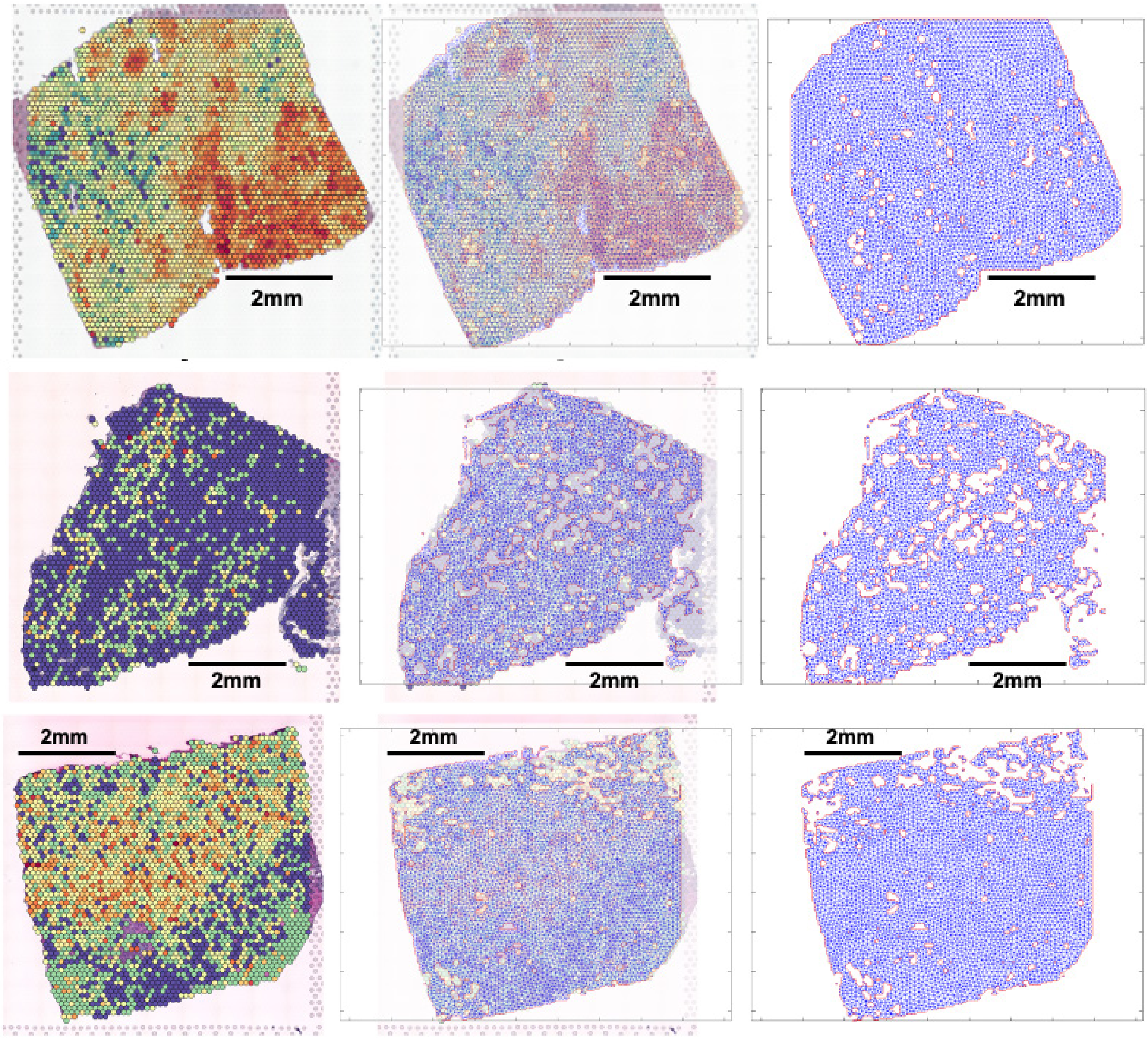

2mm
2mm
2mm
2mm
2mm
2mm
2mm
2mm
2mm

C

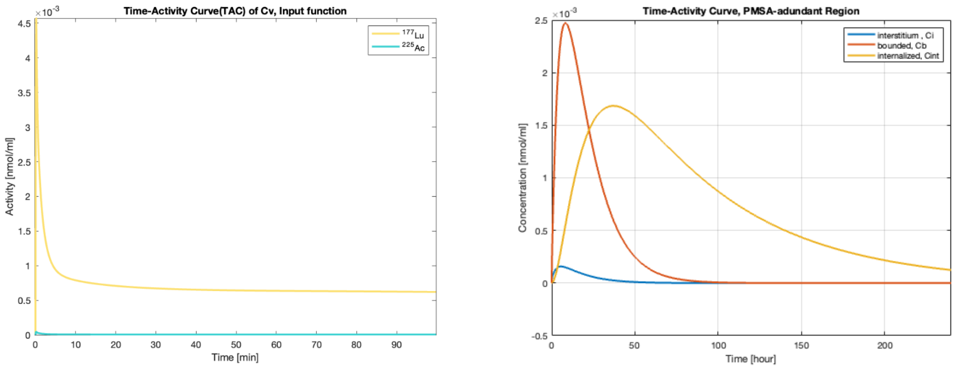

D

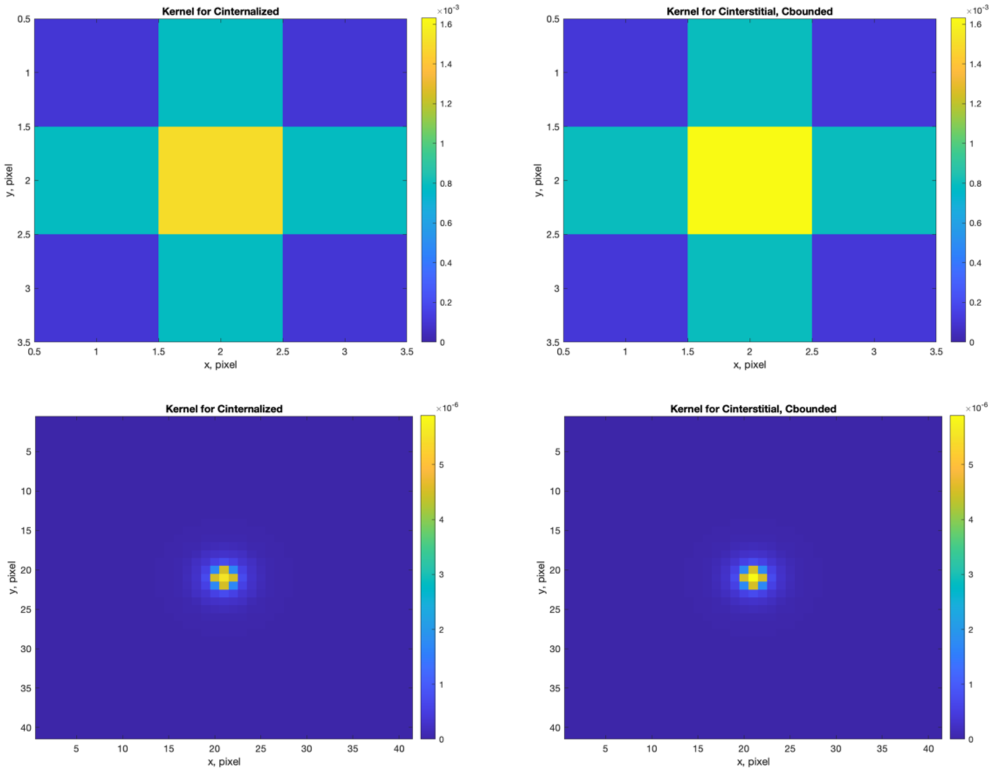

Figure S6. Solutions/mesh/kernel in Computational Domain. (A) Interstitial fluid pressure (IFP) simulated on Spatial Transcriptomics (ST) domain. **(B)** Resolution of ST and triangular mesh across three datasets. The left panel shows the ST data, the middle panel shows ST data overlaid with the triangular mesh, and the right panel displays the triangular mesh alone. Each dataset covers a 6.5 x 6.5 mm tissue area with 4992 spots per capture area, where each spot has a diameter of 55 μm and a center-to-center distance of 100 μm between spots. **(C)** Time-activity curve (TAC) of ligands in vessels (Cv) in prostate tissue (left). ^{177}Lu- and ^{225}Ac-PSMA targeting ligand are represented by yellow and blue respectively. ^{177}Lu- PSMA targeting ligand in Interstitial (Ci), Bound (Cb), and Internalized (Cint) compartments within tumor-cell rich ST spots(right). TAC for the Ci, Cb, and Cint compartments are represented by blue, orange, and yellow lines, respectively. **(D)** Dose-Point Kernel (DPK) for ^{177}Lu and ^{225}Ac PSMA-targeting RPT dose simulation. The pixel size was 40 μm s for each axis. The range of ^{225}Ac- (top) and ^{177}Lu- (bottom)-PSMA-ligand was assumed to be 1.6mm and 90μm, respectively. The kernel was made for time integrated activity of internalized compartment (left), and interstitial/bounded compartment (right).

A

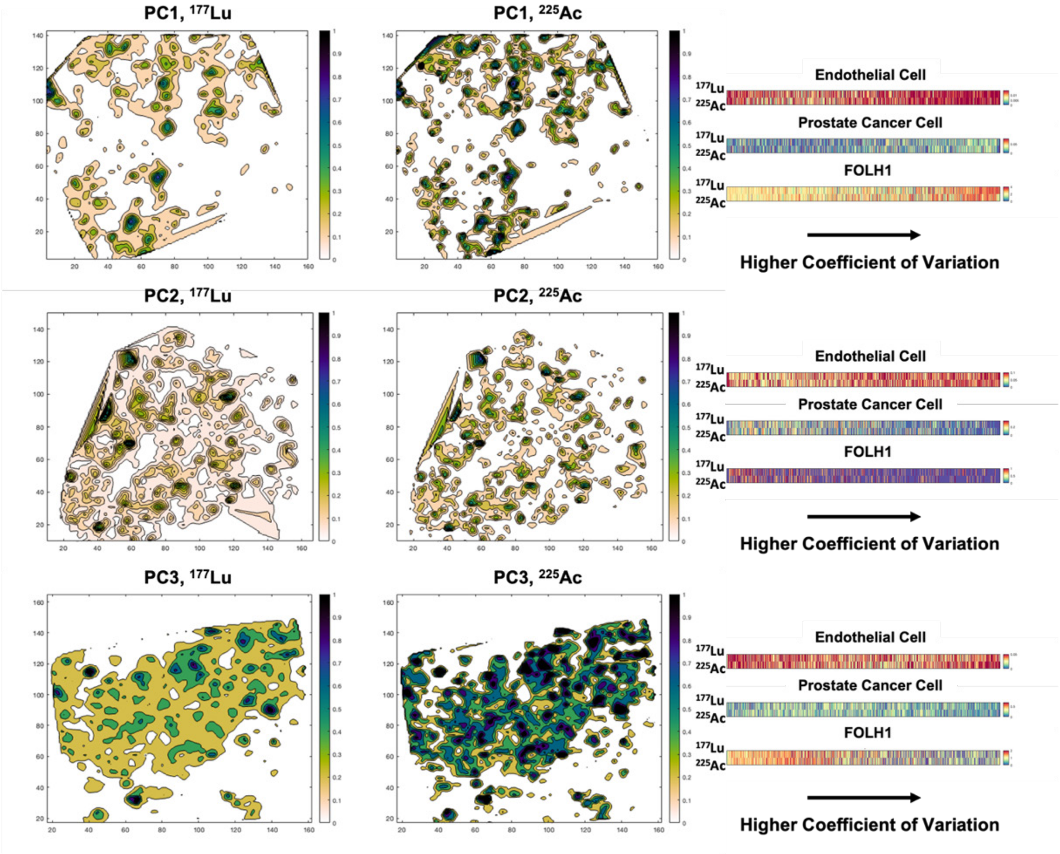

PC1, 177Lu
PC1, 225Ac
Endothelial Cell
Prostate Cancer Cell
FOLH1
Higher Coefficient of Variation
PC2, 177Lu
PC2, 225Ac
PC3, 177Lu
PC3, 225Ac

B

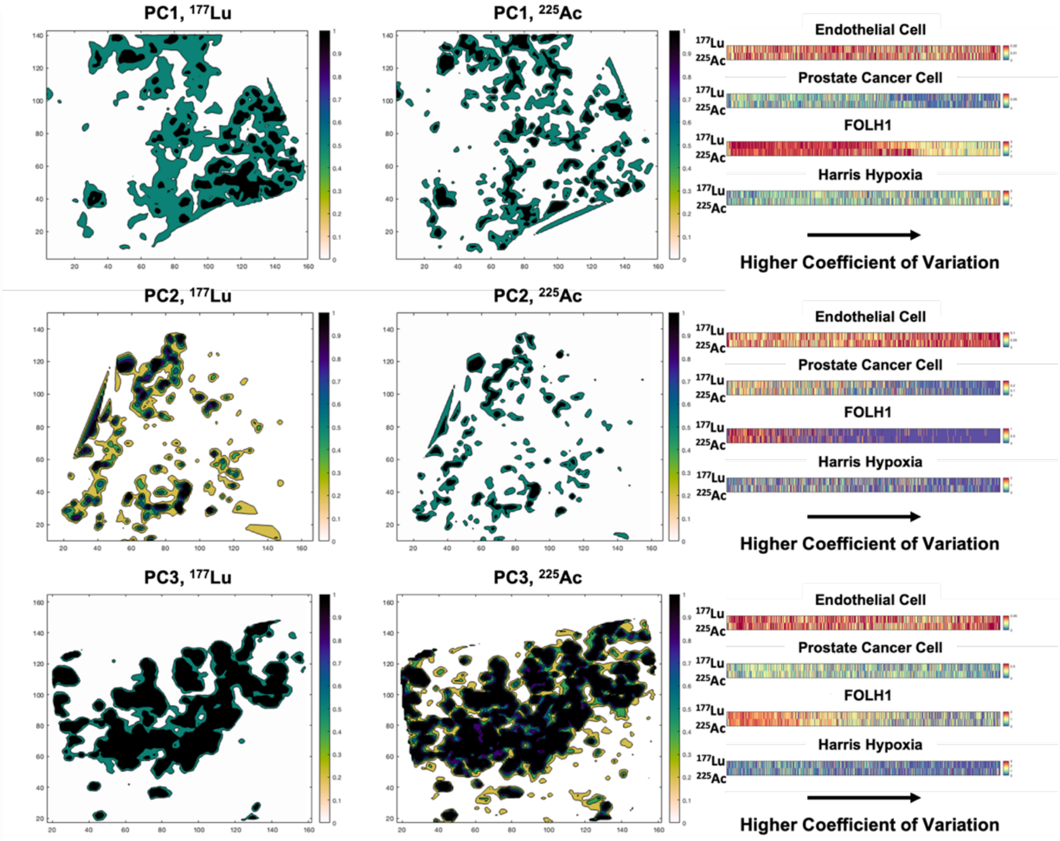

PC1, 177Lu
PC1, 225Ac
Endothelial Cell
Prostate Cancer Cell
FOLH1
Harris Hypoxia
Higher Coefficient of Variation
PC2, 177Lu
PC2, 225Ac
PC3, 177Lu
PC3, 225Ac

Figure S7. Distribution of Coefficient of Variation (CV) in Dose and Cell Survival Probability Simulation. (A) CV in Dose simulation **(B)** CV in Cell Survival Probability Simulation. The CV was calculated by iteratively applying the proposed method 20 times to assess stability. The heatmap visually represents the proportion/expression level of each endothelial cell, prostate cancer cell, and FOLH1 expression in each spatial transcriptomics (ST) spot, arranged in ascending order of CV values.

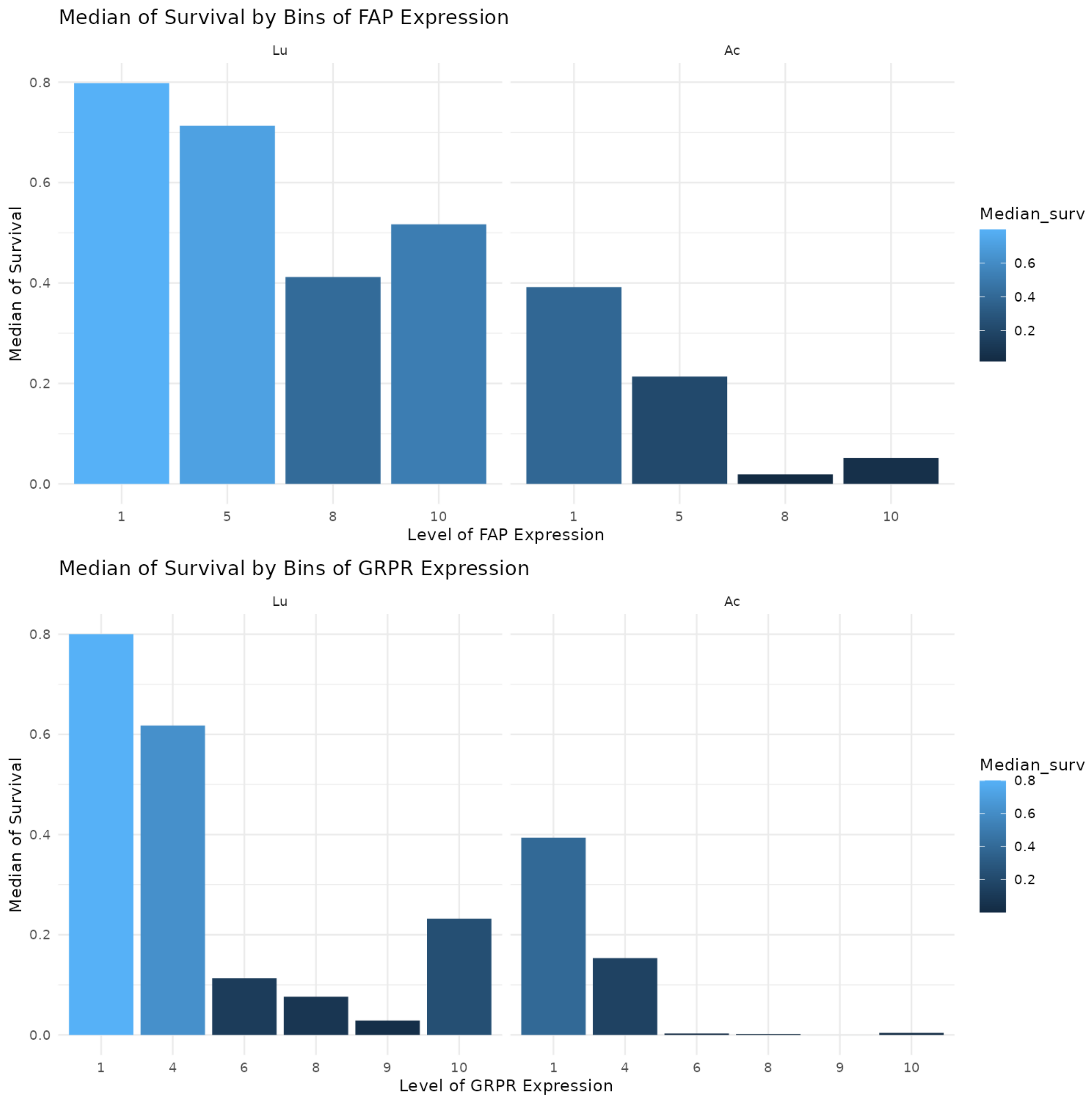

Figure S8. The bar graph displays the median cell survival values for each bin of target expression of (A) FAP or (B) GRPR, with the expression values segmented into 10 intervals. Each bin is labeled consecutively from 1 to 10.

A

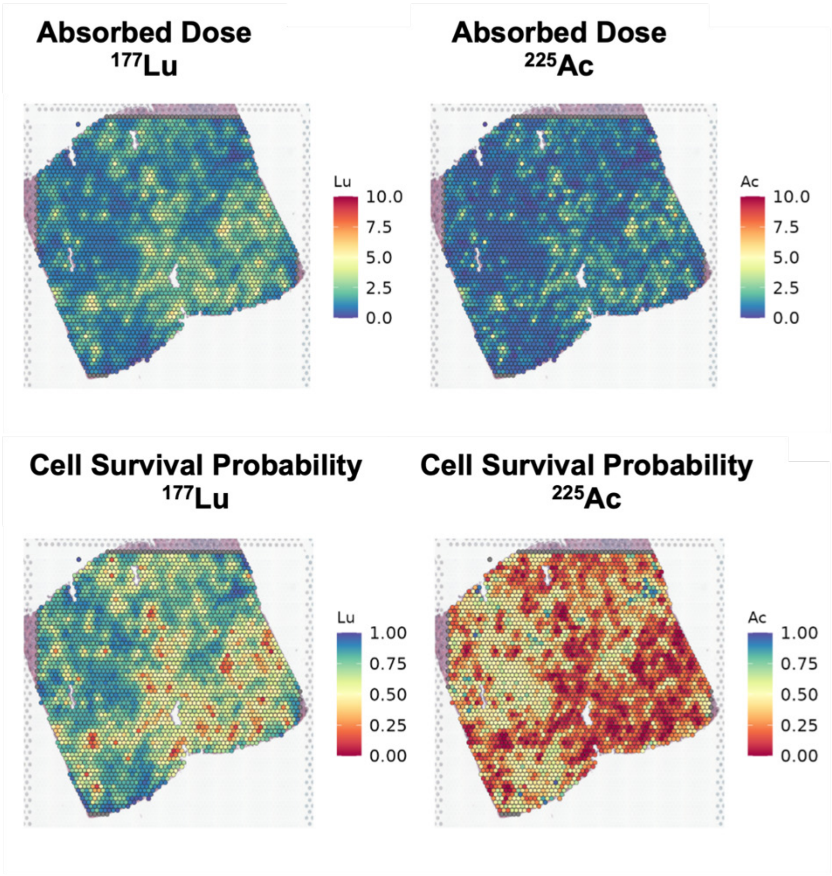
Absorbed Dose
177Lu
Absorbed Dose
225Ac
Lu
10.0
7.5
5.0
2.5
0.0
Ac
10.0
7.5
5.0
2.5
0.0
Cell Survival Probability
177Lu
Cell Survival Probability
225Ac
Lu
1.00
0.75
0.50
0.25
0.00
Ac
1.00
0.75
0.50
0.25
0.00

B

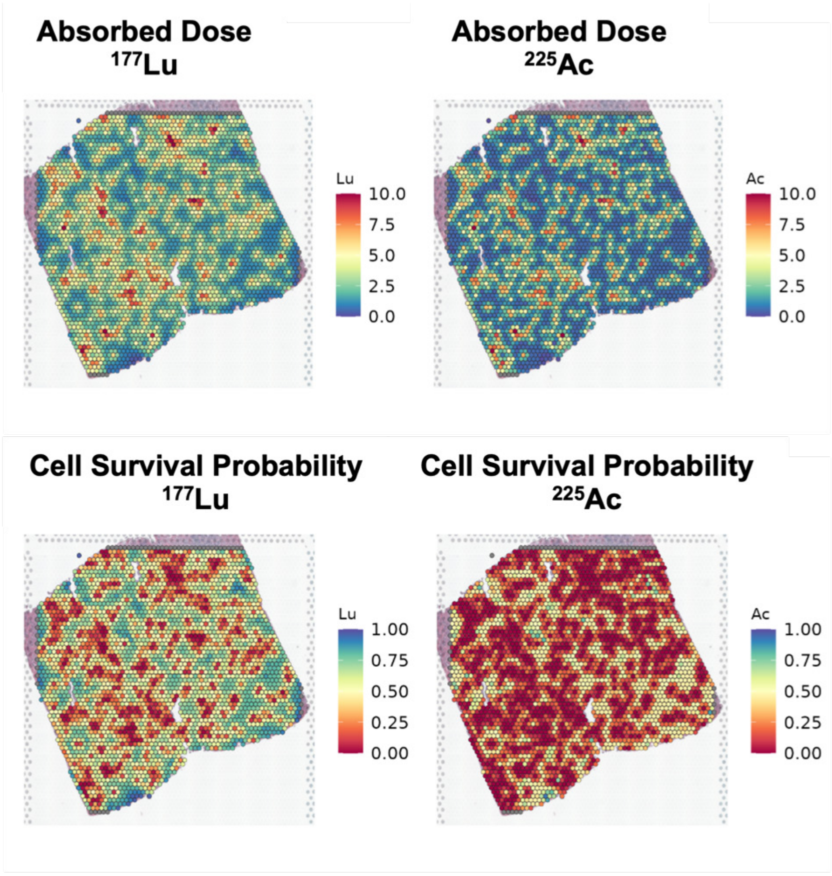
Absorbed Dose
177Lu
Absorbed Dose
225Ac
Lu
10.0
7.5
5.0
2.5
0.0
Ac
10.0
7.5
5.0
2.5
0.0
Cell Survival Probability
177Lu
Cell Survival Probability
225Ac
Lu
1.00
0.75
0.50
0.25
0.00
Ac
1.00
0.75
0.50
0.25
0.00

Figure S9. Absorbed Dose (top) and Cell Survival Probability (bottom) in ^{177}Lu (left) and ^{225}Ac (right) labelled (A) FAP – and (B) GRPR – targeted RLT. The kinetic parameters k_{off}, k_{int}, and k_{rel} are derived from the literature [53,54] assuming k_{on} is similar to that of PSMA-targeted ligand [29].

Supplementary Tables

Table S1

Conformity Indices (CI), Homogeneity Indices (HI), and Gradient Indices (GI)

Reference	Definition	^{177}Lu			^{225}Ac		
		PC1	PC2	PC3	PC1	PC2	PC3
CI_{RTOG}[63]	$CI_1 = \frac{V_{RI}}{TV}$	0.397	0.035	0.120	0.315	0.026	0.058
SALT [64]	$CI_2 = \frac{TV_{RI}}{TV}$	0.322	0.035	0.113	0.263	0.026	0.054
Van't Riet and Paddick[65]	$CI_3 = \frac{TV_{RI}}{TV} \times \frac{TV_{RI}}{V_{RI}}$	0.261	0.034	0.106	0.220	0.025	0.049
ROTG [66]	$HI_1 = \frac{D_{max}}{D_t}$	3.918	1.989	4.307	3.595	1.932	4.149
Wu et al. [67]	$HI_2 = \frac{D_2 - D_{98}}{D_t}$	3.086	1.250	2.016	2.637	1.181	1.585
Semenenko et al. [68]	$HI_3 = \frac{D_5 - D_{95}}{D_t}$	2.748	0.919	1.430	2.176	0.836	1.037
Myonggeun et al. [69]	$HI_4 = \sqrt{\sum (D_i - D_{mean})^2 \times \frac{v_i}{V}}$	59.725	13.683	22.790	43.653	11.756	15.295
$GI_{modified}$ [67]	$GI = \frac{V_{50\%}}{V_{100\%}} \times \frac{V_{RI}}{TV}$	0.485	0.154	0.273	0.429	0.114	0.162

CI, conformity index; **RTOG**, Radiation Therapy Oncology Group; **SALT**, Saint-Anne, Lariboisiere, and Tenon; $\mathbf{V_{RI}}$, the volume covered by targeted dose; **TV**: the target volume; $\mathbf{TV_{RI}}$: the target volume covered by targeted dose.

HI, homogeneity index; $\mathbf{D_{max}}$, maximum target volume; $\mathbf{D_t}$, target dose of target volume; $\mathbf{D_2}$, doses cover 2% the target volume; $\mathbf{D_5}$, doses cover 5% of the target volume; $\mathbf{D_{95}}$, doses cover 95% of the target volume; $\mathbf{D_{98}}$, doses cover 98% of the target volume; $\mathbf{D_{mean}}$, mean target volume; $\mathbf{v_i}$, volume covered by dose D_i.

GI, gradient index; $\mathbf{V_{i\%}}$**,** the volume irradiated by i% of the prescribed dose.

Table S2

Parameters used to solve interstitial fluid pressure (IFP) [60]

Symbol	Parameter	Unit	Value (normal)	Value (tumor)
K_p	Interstitial hydraulic conductivity	$cm^2 \times mmHg^{-1} \times s^{-1}$	8.5×10^{-9}	41.3×10^{-9}
p_v	Capillary hydrostatic pressure	$mmHg$	15.6	15.6
σ_T	Reflection coefficient		0.91	0.82
π_v	Capillary oncotic pressure	$mmHg$	20	20
π_i	Interstitial oncotic pressure	$mmHg$	10	15
L_p	Capillary Hydraulic permeability	$cm \times mmHg^{-1} \times s^{-1}$	0.36×10^{-7}	2.8×10^{-7}
$\left(\frac{S_b}{V_i}\right)_{avg}$	Capillary surface area per interstitial volume	cm^{-1}	800	333

Table S3

Parameters used to solve three compartments partial derivative equations (PDE) [29]

Symbol	Parameter	Unit	Value
$\boldsymbol{L_v}$	Vessel wall permeability	$cm \times s^{-1}$	3.3×10^{-4}
$\boldsymbol{D_{PSMA}}$	PSMA diffusivity	$cm^2 \times s^{-1}$	8.7×10^{-7}
$\boldsymbol{R_f}$	Modlecule/Carrier movement coefficient		1
$\boldsymbol{R_0}$	Receptor density	$nmol \times ml^{-1}$	4.089×10^{-2}
$\boldsymbol{k_{on}}$	Association rate	$ml \times nmol^{-1} \times s^{-1}$	7.7×10^{-1}
$\boldsymbol{k_{off}}$	Dissociation rate	s^{-1}	7.7×10^{-4}
$\boldsymbol{k_{int}}$	Internalization rate	s^{-1}	1.67×10^{-5}
$\boldsymbol{k_{rel}}$	Release rate	s^{-1}	2.67×10^{-6}
$\boldsymbol{FV_i}$	Fractional Interstitial volume	%	39
$\boldsymbol{FV_c}$	Fractional cellular volume	%	61
$\boldsymbol{\lambda_{dec}}$	Decay constant	s^{-1}	***^{117}Lu***: 1.197×10^{-6}<br>***^{255}Ac***: 8.087×10^{-7}
$\boldsymbol{\left(\frac{S_b}{V_i}\right)_{avg}}$	Capillary surface area per interstitial volume	cm^{-1}	333

Table S4

Parameters used for Cell Survival Probability [29]

Parameter	Physoxia	Hypoxia	Unit
$\boldsymbol{a}$	0.15	0.107	Gy^{-1}
$\boldsymbol{b}$	0.048	0.024	Gy^{-2}
$\boldsymbol{D_0}$	0.7	1.18	Gy
